# Single‐cell RNA sequencing and lipidomics reveal cell and lipid dynamics of fat infiltration in skeletal muscle

**DOI:** 10.1002/jcsm.12643

**Published:** 2020-11-27

**Authors:** Ziye Xu, Wenjing You, Wentao Chen, Yanbing Zhou, Qiuyun Nong, Teresa G. Valencak, Yizhen Wang, Tizhong Shan

**Affiliations:** ^1^ College of Animal Sciences Zhejiang University Hangzhou Zhejiang China; ^2^ Key Laboratory of Molecular Animal Nutrition (Zhejiang University) Ministry of Education Hangzhou China; ^3^ Laboratory of Animal Feed and Nutrition of Zhejiang Province Hangzhou China

**Keywords:** Fat infiltration, Sarcopenia, Intramuscular fat, Muscle wasting, Single‐cell RNA sequencing, Lipidomics, Skeletal muscle

## Abstract

**Background:**

Ageing is accompanied by sarcopenia and intramuscular fat (IMAT) infiltration. In skeletal muscle, fat infiltration is a common feature in several myopathies and is associated with muscular dysfunction and insulin resistance. However, the cellular origin and lipidomic and transcriptomic changes during fat infiltration in skeletal muscle remain unclear.

**Methods:**

In the current study, we generated a high IMAT‐infiltrated skeletal muscle model by glycerol (GLY) injection. Single‐cell RNA sequencing and lineage tracing were performed on GLY‐injured skeletal muscle at 5 days post‐injection (DPI) to identify the cell origins and dynamics. Lipidomics and RNA sequencing were performed on IMAT‐infiltrated skeletal muscle at 14 DPI (or 17 DPI for the cold treatment) to analyse alterations of lipid compositions and gene expression levels.

**Results:**

We identified nine distinct major clusters including myeloid‐derived cells (52.13%), fibroblast/fibro/adipogenic progenitors (FAPs) (23.24%), and skeletal muscle stem cells (2.02%) in GLY‐injured skeletal muscle. Clustering and pseudotemporal trajectories revealed six subpopulations in fibroblast/FAPs and 10 subclusters in myeloid‐derived cells. A subpopulation of myeloid‐derived cells expressing adipocyte‐enriched genes and Pdgfra^−^/Cd68^+^ cells displayed lipid droplets upon adipogenic induction, indicating their adipogenic potential. Lipidomic analysis revealed the changes of overall lipid classes composition (e.g. triglycerides (TAGs) increased by 19.3 times, *P* = 0.0098; sulfoquinovosyl diacylglycerol decreased by 83%, *P* = 0.0056) and in the distribution of lipids [e.g. TAGs (18:2/18:2/22:6) increased by 181.6 times, *P* = 0.021] between GLY‐group and saline control. RNA‐seq revealed 1847 up‐regulated genes and 321 down‐regulated genes and significant changes in lipid metabolism‐related pathways (e.g. glycerolipid pathway and glycerophospholipid pathway) in our model of GLY‐injured skeletal muscle. Notably, short‐term cold exposure altered fatty acid composition (e.g. saturated fatty acid decreased by 6.4%, *P* = 0.058) in fat‐infiltrated muscles through directly affecting lipid metabolism pathways including PI3K–AKT and MAPK signalling pathway.

**Conclusions:**

Our results showed that a subpopulation of myeloid‐derived cells may contribute to IMAT infiltration. GLY‐induced IMAT infiltration changed the lipid composition and gene expression profiles. Short‐term cold exposure might regulate lipid metabolism and its related signalling pathways in fat‐infiltrated muscle. Our study provides a comprehensive resource describing the molecular signature of fat infiltration in skeletal muscle.

## Introduction

Muscle fat infiltration, a common feature of several myopathies, increases during the ageing process.^1^, ^2^, ^3^ Increased adipocyte infiltration and fat deposition in skeletal muscle were also observed in several diseases and during physiological stress.^4^, ^5^, ^6^, ^7^ Recent studies have reported that excessive intermuscular fat accumulation decreases muscle strength^4^ and induces insulin sensitivity^8^ and disturbances in lipid metabolism,^9^ suggesting that intramuscular fat (IMAT, all abbreviations were provided in Supporting Information, *Table* S1) is associated with muscular dysfunction. As a pathological characteristic for several diseases, the local IMAT could have a feedback effect on the whole‐body metabolism and may be significantly correlated with other diseases, such as cardiovascular disease.^10^ Interestingly, in meat production, a high content of IMAT is positively correlated with meat quality traits, such as tenderness and juiciness.^11^ Considering the ubiquity of IMAT and its association with muscular dysfunction and related diseases as well as meat quality, it is necessary to understand the regulatory mechanisms of IMAT infiltration and their impact on lipid metabolism in skeletal muscle, to subsequently develop innovative therapies combatting these pathological conditions as well as for improving meat quality.

Several investigations have been recently carried out to define the origin of IMAT and demonstrated that several cell types contribute to ectopic IMAT formation. Fibro/adipogenic progenitors (FAPs), which are mainly positive for the cell surface marker platelet‐derived growth factor receptor alpha (*Pdgfra* or *Cd140a*), proliferate and differentiate into adipose and/or fibrous tissue in pathological conditions.^3^, ^12^ Some pathological conditions, other physiological processes, and pharmacological treatments could induce the rate of muscle‐derived stem cells entering the adipogenic lineage.^13^, ^14^, ^15^ Several pathways and factors (peroxisome proliferator‐activated receptors, WNT growth factors, myokines, mitochondrial reactive oxygen species production, and protein kinase C beta) have been identified to be involved in the adipogenic conversion of satellite cells.^13^ A stationary population of progenitor cells expressing CD34 surface protein or expressing myogenic factor 5 (Myf5) in skeletal muscle has a high potential for *in vitro* differentiation into genuine brown adipocytes.^16^ Because skeletal muscles are highly heterogeneous and contain several cell types (myogenic, adipogenic, endothelial, and immune cells), it needs to be clarified whether other kinds of cells might contribute to IMAT formation.

Previous assumptions identified IMAT as white adipose and that uncoupling protein 1 (UCP1) expression was hardly detectable in normal skeletal muscles. However, brown adipocytes in skeletal muscles have been recently identified.^17^ UCP1 expression was induced during adipocyte infiltration in skeletal muscle and influenced rates of energy expenditure while being controlled both genetically and hormonally.^18^, ^19^ We hypothesized that by experimentally induced cold exposure, a shifting of intramuscular adipocytes towards a brown phenotype may be functionally related to some improvements in systemic metabolism. Cold exposure has been reported to be an efficient method to induce energy expenditure by activating UCP1 and influencing the dynamics of lipid metabolism and transcriptional processes in brown and white adipose tissue.^20^, ^21^ However, lipidomic alteration and its regulatory mechanism in the adipocyte‐infiltrated skeletal muscle remain unclear.

Previous studies reported a similarity between the intramuscular glycerol (GLY) injection‐induced degenerative changes and those recorded in Duchenne muscular dystrophy (DMD) (such as myofibre hypercontraction, plasma membrane disruption, vacuolar changes, variation in fibre size, selective loss of Z‐bands, and followed ectopic adipocyte infiltration). They concluded that experimental GLY‐induced myopathy could be a suitable model to study the pathophysiology of DMD.^22^ In order to reveal the adipocyte origin as well as the precise lipidome and transcriptomic changes of adipocyte infiltration in skeletal muscle, we generated a high‐IMAT infiltration mouse model by intramuscular GLY injection. Based on the reported timeline of recruited and activated skeletal muscle‐resident cells,^22^ we collected mononuclear cells at 5 days post‐injection (DPI) with relatively more recruited or activated mononuclear cells, especially adipose‐derived stem cells and sampled GLY‐injected muscle at 14 DPI (17 DPI for the cold treatment) with regenerated myotubes and large‐scale ectopic adipocyte infiltration, and applied them to single‐cell RNA sequencing (scRNA‐seq), lipidomics, and RNA sequencing, respectively, to provide a comprehensive resource describing the cell origins and lipidomic and transcriptomic profiles of IMAT infiltration in skeletal muscle. Lipidomics and transcriptomics were also applied to reveal potential effects of cold exposure on lipid metabolism of the high‐IMAT infiltration model. Our findings provide novel insights into understanding the molecular signature of fat infiltration in skeletal muscles, which may become important for the development of therapies to combat fat infiltration‐related myopathies and diseases.

## Materials and methods

### Animals

All the procedures involving mice were approved by Zhejiang University Animal Care and Use Committee. Male C57BL6/J mice were single housed under standard laboratory conditions, including a 12 h light/dark cycle, with free access to mouse diet and water. For the scRNA‐seq experiment, 10 adult wild‐type mice were anaesthetized with an intraperitoneal injection of pentobarbital sodium, 0.02 mg per body weight (g). The anterolateral part of tibialis anterior (TA) muscle was shaved, and 100 μL of 50% GLY (v/v) in sterile 0.9% NaCl was injected along the length of the TA muscle as previously described.^23^ Each animal was kept on a heating pad at approximately 38°C to maintain body temperature until full recovery. Based on the reported timeline of recruited and activated skeletal muscle‐resident cells,^22^ mice were sacrificed after 5 DPI, then TA muscles were sampled, and immediately subjected to single‐cell isolation and scRNA‐seq. For the lipidomics (*n* = 8) and bulk RNA‐seq (*n* = 4) and other experiments, TA muscle was treated as previously described and sampled at 14 DPI. For the cold treatment experiment, these GLY‐injected mice (14 DPI) were housed at either room temperature (RT) or COLD (4°C) for 3 days and were subsequently used for tissue sampling. The whole TA samples were carefully sampled, frozen immediately in liquid nitrogen, and stored at −80°C for the subsequent analyses. For the lineage tracing experiment, *Pdgfra‐Cre* ^*ER*^ (Stock No. 018280) and *ROSA* ^*mT/mG*^ (Stock No. 007676) mice were purchased from Jackson Laboratory (Bar, Harbor, MA, USA), and *Pdgfra‐mT/mG* mice were generated. TA muscles were injected with GLY and sampled and immediately subjected to single‐cell isolation as described previously.

### Single‐cell RNA‐seq using 10x genomics chromium

We pooled samples from 10 experimentally treated mice and performed scRNA‐seq of all alive cells isolated from GLY‐injected TA muscles. GLY‐injected TA muscles were dissociated with enzymatic digestion with collagenase I for 30 min at a concentration of 0.15 g per 100 mL and filtrated by cell sieve (40 μm). Isolated single cells were applied to Red Blood Cell Lysis Buffer and the magnetic bead separation method to remove red blood cells and dead cells. The viability of filtrated single cells was assessed via trypan blue (Thermo Fisher Scientific, waltham, MA, USA) and using a haemocytometer (Thermo Fisher Scientific). Following counting, the appropriate volume was calculated for a target capture of 7000 cells. Samples below the required cell concentration as defined by the user guide (700–1200 cells/μL) were pelleted and resuspended in a reduced volume and counted again using a haemocytometer prior to loading onto the 10x Genomics single‐cell‐A chip. Reverse transcription and library preparation were performed using the 10x Genomics Single Cell v2 kit following the 10x Genomics protocol. The library was multiplexed and sequenced on one lane of Illumina NextSeq‐500 with a high‐output (400 m) kit.

Quality control of 10x Genomics single‐cell RNA‐seq was conducted. For mapping, sequences obtained from sequencing using the 10x Genomics single‐cell RNA‐seq platform were demultiplexed and mapped to the mm10 transcriptome using the Cell Ranger package (10x Genomics). Next, the raw digital gene expression matrix (UMI counts per gene per cell) obtained from Cell Ranger package analyses was filtered, normalized by using the R package Seurat (Version 2.3.4).^24^ The cells were removed if they expressed fewer than 500 unique genes, more than 50 000 UMI counts, or greater than 10% mitochondrial reads. Those genes that were not detected in any cell were removed from subsequent analysis. The number of cells after filtration in current study was 5782.

### t‐Distributed stochastic neighbour embedding analysis of single‐cell RNA‐seq datasets and identification of cell clusters

After log normalizing the data, a principal component analysis (PCA) was performed to reduce dimension. The identification of significant clusters was performed using the Find Clusters algorithm in the Seurat package, which uses a shared nearest‐neighbour modularity optimization‐based clustering algorithm. Marker genes for each significant cluster were found using the Seurat function FindAllMarkers. Cell types were determined using a combination of marker genes identified from the literature and gene ontology for cell types. Expression of selected genes was plotted with the Seurat function FeaturePlot and VlnPlot. Hierarchical clustering and heat map generation were performed for single cells based on log‐normalized (with scale factor 10 000 and pseudocount 1) expression values of marker genes obtained from the literature or identified as highly differentially expressed. Heat maps were generated using the heatmap.2 function from the gplots v3.6.1 R package using the default complete‐linkage clustering algorithm.

### Pseudotime trajectory analysis

Pseudotime trajectory was plotted by using the R package monocle Version 2.4 with the default settings given there. Pseudotime ordering was performed using the function ‘reduce dimension’ with max_components set at 2 and reduction_method set as DDRTree. Next, the significantly affected genes were obtained from the top 50 markers among the clusters by using the function differentialGeneTest (fullModelFormulaStr = ~Pseudotime) and were plotted with the function plot_pseudotime_heatmap. The num_cluster was set at 4 to obtain four modules of significantly changed genes that had similar trends according to their pseudotemporal expression patterns.

### Haematoxylin–eosin staining

TA muscles from mice were fixed in 10% formalin for 24 h at RT. Then the tissues were embedded into paraffin, blocked, and cut at 5–10 μm for haematoxylin–eosin (H&E) staining. The sections were deparaffinized, rehydrated, and stained with haematoxylin for 15 min. Sections were then rinsed in running tap water and stained with eosin for 3–5 min, dehydrated, mounted, and captured.

### Total RNA extraction and quantitative real‐time PCR

Total RNA extraction and real‐time PCR were performed as previously described.^25^ Briefly, total RNA was extracted from TA muscles using TRIzol reagent (Thermo Fisher Scientific), and the purity and concentration of total RNA were measured. Two micrograms of total RNA was reverse transcribed using random primers and MMLV reverse transcriptase (Thermo Fisher Scientific). Real‐time PCR was carried out with an Applied Biosystems StepOnePlus™ Real‐Time PCR System using SYBR Green Master Mix (Roche, Indianapolis, IN, USA) and gene‐specific primers (*Table* S2). The 2^−ΔΔCT^ method was used to analyse the relative changes in gene expression normalized against 18S ribosomal RNA as an internal control.

### Magnetic cell sorting

After removing red blood cells and dead cells, the isolated single cells were applied to magnetic cell sorting (MACS) according to the method of Korkusuz *et al*.^26^ Pdgfra^+^ cells and Pdgfra^−^ cells were separated using magnetic microbeads conjugated with anti‐Pdgfra (CD140a) antibody (CD140a antibody, anti‐mouse, 130‐101‐905, Miltenyi Biotec, Bergisch Gladbach, Germany). Pdgfra^−^/Cd68^+^ and Pdgfra^−^/Cd68^−^ cells were further sorted from Pdgfra^−^ cells using magnetic microbeads conjugated with anti‐CD68 antibody (CD68 antibody, anti‐mouse, 130‐102‐585, Miltenyi Biotec). For single cells isolated from *Pdgfra‐mT/mG* mice, cells were directly incubated with magnetic microbeads conjugated with anti‐CD68 antibody to separate CD68^+^ and CD68^−^ cells.

### Adipogenic differentiation

Cells sorted by MACS were cultured in Dulbecco's modified Eagle's medium containing 20% foetal bovine serum for 1 day and then exposed to adipogenic induction medium with the adipogenic differentiation agent, MDI (0.5 mM 3‐isobutyl‐1‐methylxanthine, 1 μM dexamethasone, and 1 mg/L insulin), for 3 days. The medium was replaced with maintenance medium containing 1 mg/L insulin and 10% foetal bovine serum–Dulbecco's modified Eagle's medium until Day 5, and fresh medium was added every 2 days.

### Lipid sample preparation and lipidomic assay

Lipid extraction and mass spectrometry‐based lipid detection were performed by Applied Protein Technology Company. We took a separate sample from each group and mixed them equally together to create a pooled quality control sample. Quality control samples were inserted into the analysis queue to evaluate system stability and data reliability during the whole experimental process. Liquid chromatography–mass spectrometry/mass spectrometry analysis was performed on a Q Exactive plus mass spectrometer (Thermo Scientific) coupled to a UHPLC Nexera LC‐30A (Shimadzu, Kyoto, Japan). Full‐scan spectra were collected in mass‐to‐charge ratio (*m*/*z*) ranges of 200–1800 and 250–1800 for positive‐ion and negative‐ion modes, respectively. The mass‐to‐charge ratio of lipid molecules to lipid fragments was collected by the following method: after each full scan, 10 fragment patterns (MS2 scan, HCD) were collected. Lipid identification (secondary identification), peak extraction, peak alignment, and quantification were assessed with the LipidSearch software Version 4.1 (Thermo Scientific™). In the extracted ion features, only the variables having more than 50% of the non‐zero measurement values in at least one group were kept.

### Unsupervised multivariate data analyses

For the multivariate statistical analysis, the SIMCA‐P 14.1 software (Umeta, Umeå, Sweden) was used. After the Pareto scaling, a PCA and a partial least squares discriminant analysis were performed. The leave‐one‐out cross‐validation and response permutation testing were used to evaluate the robustness of the model. The significantly different components were determined based on the combination of a statistically significant threshold of variable influence on projection (VIP) values obtained from partial least squares discriminant analysis model and two‐tailed Student's *t*‐test (*P*‐value) on the raw data. Those metabolites with VIP values larger than 1.0 and *P*‐values less than 0.05 were considered as significant. Univariate analysis included the Student's *t*‐test and variable fold‐change analysis. Hierarchical cluster analysis and correlation analysis were performed with R software (Version 3.5.1). All the statistical evaluations using the PCA and orthogonal projections to latent structures (OPLS) methods described in this work were calculated from relative abundances.

### RNA‐seq analysis

RNA extraction and RNA‐seq analysis were performed by Sangon Biotech (Shanghai, China). Next, total RNA was extracted using the Total RNA Extractor (TRIzol) kit (B511311, Sangon Biotech) according to the manufacturer's protocol, and it was treated with RNase‐free DNase I to remove genomic DNA contamination. A total amount of 2 μg RNA per sample was used as input material for the RNA sample preparations. Sequencing libraries were generated using VAHTSTM mRNA‐seq V2 Library Prep Kit for Illumina^®^, following the manufacturer's recommendations, and index codes were added to attribute sequences to each sample. The libraries were then quantified and pooled. Paired‐end sequencing of the library was performed on the HiSeq XTen sequencers (Illumina, San Diego, CA). FastQC (Version 0.11.2) was used for evaluating the quality of sequenced data. Raw reads were filtered by Trimmomatic (Version 0.36). Clean reads were mapped to the reference genome by HISAT2 (Version 2.0) with default parameters. RSeQC (Version 2.6.1) was used to run statistics on the alignment results. The homogeneity distribution and the genomic structure were checked by Qualimap (Version 2.2.1). BEDTools (Version 2.26.0) was used for statistical analysis of the gene coverage ratio. Gene expression values of the transcripts were computed by StringTie (Version 1.3.3b). The transcripts per kilobase million (TPM) eliminated the influence of gene lengths and sequencing discrepancies to enable direct comparison of gene expression between samples. DESeq2 (Version 1.12.4) was used to determine differentially expressed genes (DEGs) between two samples. Genes were considered as significantly different expressed if *q*‐value <0.001 and |FoldChange| > 1.5.

### Pathway enrichment analysis

Functional enrichment analyses, including Gene Ontology (GO) and Kyoto Encyclopedia of Genes and Genomes (KEGG), were used to identify which DEGs were significantly enriched in GO terms or metabolic pathways. GO is an international standard classification system for gene function. DEGs are mapped to the GO terms (biological functions) in the database. The number of genes in each term was calculated, and a hypergeometric test was performed to identify significantly enriched GO terms in the gene list out of the background of the reference gene list. GO terms and KEGG pathways with false discovery rates *P* < 0.05 were considered as significantly different.

### Data analysis

All the statistical analyses of the lipidomic data described in this work were calculated from relative abundances. Experimental data are presented as the mean ± standard error of the mean. Comparisons were made by unpaired two‐tailed Student's *t*‐tests or one‐way analyses of variance, as appropriate. Differences between groups were considered statistically significant at *P* < 0.05.

## Results

### scRNA‐seq identified distinct cell populations in glycerol‐injured skeletal muscle

**Figure 1:**
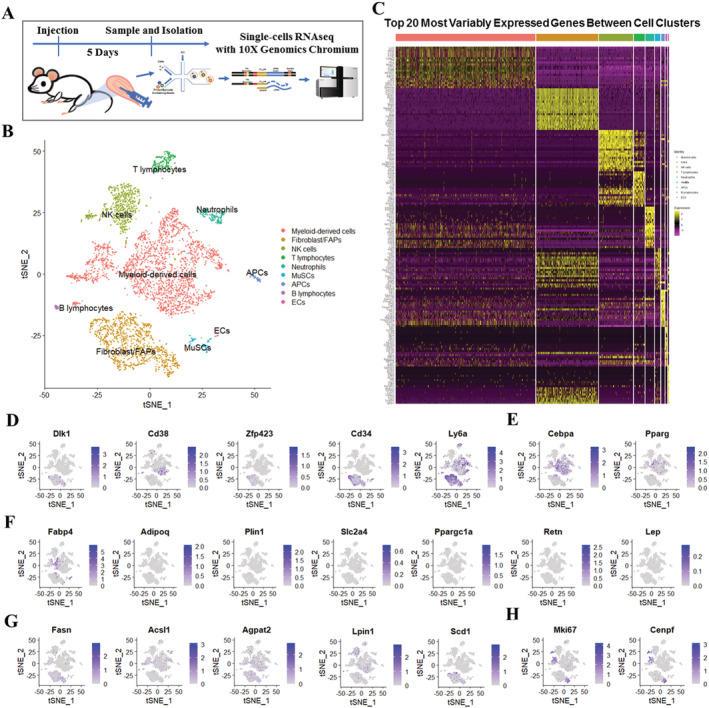
scRNA‐seq identifies distinct cell populations in GLY‐injured skeletal muscle. (A) Scheme of muscle preparation, single‐cell isolation, and scRNA‐seq at 5 DPI. (B) Graph‐based clustering of isolated single cells identifies distinct clusters corresponding to different cell populations. (C) Heat map representing the top 20 most differently expressed genes between cell clusters identified. Colours and numbers correspond to the cell clusters shown in (B). Expression of (D) preadipocyte‐enriched genes (*Dlk1*, *Cd38*, *Zfp423*, *Cd34*, and *Ly6a*), (E) adipogenic master regulators (*Cebpa* and *Pparg*), (F) late adipogenic genes (*Fabp4*, *Adipoq*, *Plin1*, *Slc2a4*, *Ppargc1a*, *Retn*, and *Lep*), (G) lipogenic genes (*Fasn*, *Acsl1*, *Agpat2*, *Lpin1*, and *Scd1*), and (H) proliferation genes (*Mki67* and *Cenpf*) in these populations.

To characterize the cell origins and dynamics of adipocyte infiltration in skeletal muscle, we generated a GLY injection‐induced skeletal muscle injury model. H&E histological views showed disrupted myofibres and regenerated myofibres with centre nuclear surrounded by infiltrating cells in GLY‐injected TA at 5 DPI (*Figure* S1A). Mononuclear cells were isolated from the GLY‐injured TAs at 5 DPI and applied to scRNA‐seq analysis (*Figure* 1A). The results obtained from Cell Ranger analyses were shown in *Figure* S1B. The estimated number of cells in the current study was 6031. Fraction reads in cells were 95.6%. Mean reads per cell were 49 446. Median genes per cell were 2901. Total genes detected were 27 923. Median UMI counts per cell were 10 180 (*Figure* S1B). Following the quality control of scRNA‐seq data, we retained 5782 cells for the downstream analysis (*Figure* S1C and S1D). Aggregated and normalized scRNA‐seq data were then subjected to unsupervised graph‐based clustering to identify the cell types, which were projected onto the t‐distributed stochastic neighbour embedding (t‐SNE) plots using Seurat R Package (*Figure* 1B).^24^ Our analysis using a combination of marker genes identified nine putative major cell types, including myeloid‐derived cells (52.13%), fibroblast/FAPs (23.24%), natural killer cells (NK cells) (12.73%), T lymphocytes (4.08%), neutrophils (3.34%), skeletal muscle stem cells (MuSCs) (2.02%), antigen‐presenting cells (APCs) (1.30%), B lymphocytes (0.71%), and endothelial cells (ECs) (0.45%) (*Figures* 1B and S1E). Visualization of the top 20 most variably expressed genes between cell clusters showed distinct transcriptional programs of the nine clusters (*Figure* 1C and *Table* S3) and expression of known cell lineage‐enriched or cell lineage‐specific transcripts in each of the cell clusters, including myeloid‐derived cells (*Cd68*), fibroblast/FAPs (*Pdgfra*), NK cells (*Gzma*), T lymphocytes (*Cd28*), neutrophils (*Cd14*), MuSCs (*Myod1*), APCs (*H2‐Eb1*), B lymphocytes (*Cd19*), and ECs (*Pecam1*) (*Figure* S1F). To predict the origin of adipocytes infiltrated during muscle regeneration, we examined the expression of a list of genes identified as adipogenesis‐related and lipogenesis‐related genes.^27^, ^28^, ^29^ The expression patterns of preadipocyte‐enriched genes (*Dlk1*, *Cd38*, *Zfp423*, *Cd34*, and *Ly6a*), adipogenic master regulators (*Cebpa* and *Pparg*), late adipogenic genes (*Fabp4*, *Adipoq*, *Plin1*, *Slc2a4*, *Ppargc1a*, *Retn*, and *Lep*), lipogenic genes (*Fasn*, *Acsl1*, *Agpat2*, *Lpin1*, and *Scd1*), and proliferation genes (*Mki67* and *Cenpf*) in these populations were analysed (*Figure* 1D–1H). Notably, preadipocyte‐enriched genes (*Cd38*, *Zfp423*, *Cd34*, and *Ly6a*), adipogenic master regulators (*Cebpb* and *Pparg*), late adipogenic gene (*Fabp4*), and lipogenic genes (*Fasn*, *Acsl1*, *Agpat2*, *Lpin1*, and *Scd1*) showed wide expression in clusters of fibroblast/FAPs and myeloid‐derived cells (*Figure* 1D–1G). Proliferation genes (*Mki67* and *Cenpf*) showed higher expression levels in parts of the myeloid‐derived cell clusters, NK cells, and FAPs (*Figure* 1H). There was hardly any expression of mature adipocyte differentiation markers (*Adipoq*, *Plin1*, *Slc2a4*, *Ppargc1a*, *Retn*, and *Lep*) in all clusters (*Figure* 1F). These adipogenesis‐related and lipogenesis‐related gene expression patterns suggest that both fibroblast/FAPs and myeloid‐derived cell clusters might participate in the fat deposition during GLY‐induced muscle regeneration.

### Clustering and pseudotemporal trajectories identified transcriptional dynamics of fibroblast/fibro/adipogenic progenitors

**Figure 2:**
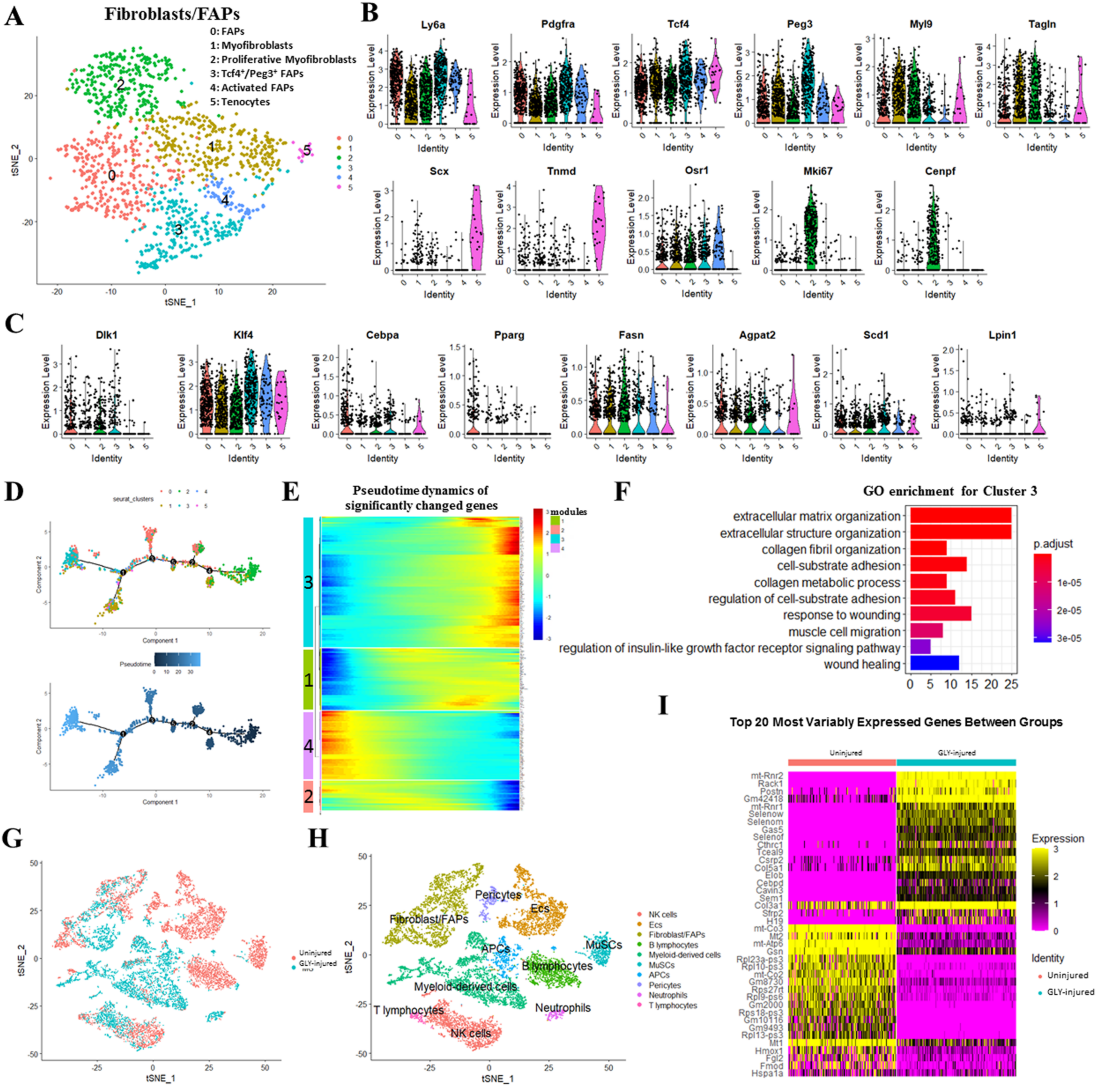
Clustering and pseudotemporal trajectories identify transcriptional dynamics of fibroblast/FAPs. (A) Graph‐based clustering of fibroblast/FAPs showing six subclusters. (B) Expression of marker genes (*Ly6a*, *Pdgfra*, *Tcf4*, *Peg3*, *Myl9*, *Tagin*, *Scx*, *Tnmd*, *Osr1*, *Mki67*, and *Cenpf*). (C) Expression of adipogenesis‐related markers (*Dlk1*, *Klf4*, *Cebpa*, *Pparg*, *Fasn*, *Agpat2*, *Scd1*, and *Lpin1*). (D) Pseudotime single‐cell trajectory reconstructed by Monocle2 for fibroblast/FAPs. Pseudotime is shown coloured in a gradient from dark to light blue, and start of pseudotime is indicated. (E) Pseudotemporal heat map showing gene expression dynamics for significant marker genes. Genes (rows) were clustered into four modules, and cells (columns) were ordered according to pseudotime. (F) GO enrichment analysis of genes in Module 4. (G) The t‐SNE plot of merged isolated single cells forms normal and GLY‐injured skeletal muscle. (H) Graph‐based clustering of merged isolated single cells forms normal and GLY‐injured skeletal muscle. (I) Heat map of top 20 significant genes between normal and GLY‐injured fibroblast/FAPs.

Of the total clusters, fibroblasts/FAPs revealed a gene expression pattern that could be assigned to adipocyte‐derived stem cells. Further analysis based on shared nearest‐neighbour clustering using the single‐cell R toolkit Seurat^24^ on fibroblast/FAPs returned six juxtaposed subclusters (*Figure* 2A). Subclusters 0 and 3 expressed relatively higher levels of *Ly6a* and the univocal gene marker of FAPs, *Pdgfra* (*Figure* 2B). Subcluster 3 also shows overlap with Tcf4^+^ cells originally defined as muscle connective tissue fibroblasts, as well as with the Pdgfra^+^ subpopulation of PW1^+^ (*Peg3*) interstitial cells (*Figure* 2B). Subclusters 1 and 2 expressed relatively higher levels of myofibroblast markers (*Myl9* and *Tagln*) (*Figure* 2B). Cells in Subcluster 2 were proliferative as they expressed higher levels of *Mki67* and *Cenpf* (*Figure* 2B). Consistent with this, cell cycle analysis based on mouse cell cycle markers revealed that most of G_2_/M‐stage cells were located in Subcluster 2 (*Figure* S2A and S2B). Most of the cells in Subcluster 4 expressed the activated FAP marker (*Osr1*) (*Figure* 2B). Subcluster 5 was marked by the expression of tenocyte markers (*Scx* and *Tnmd*) (*Figure* 2B). Early adipogenic genes (*Dlk1* and *Klf4*) had relatively higher expression level in Subcluster 3 (*Figure* 2C). Later adipogenic genes (*Cebpa* and *Pparg*) had relatively higher expression level in Subcluster 0 (*Figure* 2C). A fraction of cells in Subcluster 0 expressed *Fabp4*, while these other full differentiation markers (*Adipoq*, *Plin1*, *Lep*, and *Slc2a4*) virtually showed no expression (*Figure* S2C). Pseudotime trajectory analysis was used to analyse progression of continuous cell states of fibroblast/FAPs and revealed ordered cells expressing different levels of marker genes in a trajectory (*Figures* 2D and S2D). Ordering of cells in pseudotime arranged most of the fibroblast/FAPs into a major trajectory, with five bifurcations and 11 stages (*Figures* 2D, S2D, and S2E). Proliferative Subcluster 2 was located towards the origin of the trajectory, which partly served as a validation for the constructed trajectory (*Figures* 2D and S2E). Early (Subcluster 3) and later (Subcluster 0) adipogenic FAPs were located towards the terminus of the bifurcations of the trajectory, and the myofibroblasts (Subcluster 1) were located towards the other terminal bifurcation of the trajectory (*Figures* 2D and S2E). We also examined pseudotime dynamics of significantly changed genes among these six subclusters and arranged them into four modules according to their pseudotemporal expression patterns (*Figure* 2E and *Table* S4). GO analysis of genes in Module 3, which followed similarly up‐regulated kinetic trends, revealed clear enrichment in the extracellular matrix organization and collagen fibril organization, without involvement of the adipogenesis‐related pathways (*Figure* 2F). This is consistent with previous reports that the basal lamina was disrupted in GLY‐injured model and ectopic adipocyte infiltration was detected by at least 7 days after GLY injection.^23^, ^30^, ^31^

To compare the transcriptome programs of fibroblast/FAPs in non‐injured and GLY‐injured skeletal muscles, we computationally merged that of Giordani *et al*.^32^ with our scRNA‐seq datasets (*Figure* 2G) and the housekeeping genes (*Vcp*, *Psmb2*, and *Psmb4*) and observed similar expression levels after calibrating batch effects (*Figure* S2F). The analysis of the experimentally merged scRNA‐seq datasets based on shared nearest‐neighbour clustering returned a total of 10 cell types (*Figure* 2H). Consistent with our previous results, fibroblast/FAPs from GLY‐injured muscle were remarkably higher expressed in the genes related to collagen (*Col5a1*, *Col3a1*, and *Cthrc1*) and extracellular matrix (*Postn*) development. Mitochondria‐related (*mt‐Co3*, *mt‐Atp6*, and *mt‐Co2*) and ribosome‐related (*Rpl23a‐ps3*, *Rpl10‐ps3*, *Rps27rt*, *Rpl9‐ps6*, *Rpl18‐ps3*, and *Rpl13‐ps3*) genes showed lower levels in GLY‐injured muscles (*Figure* 2I). Furthermore, Kuang's scRNA‐seq datasets of non‐injured and 5 DPI‐cardiotoxin (CTX)‐injured muscles were also merged into ours with calibrating batch effects and shared nearest‐neighbour clustering analysis returned a total of 11 cell types (*Figure* S2G–S2I). Consistently, collagen (*Col8al*, *Col12al*, *Cthrc1*, and *Col16a1*) and extracellular matrix (*Postn* and *Mfap4*) development‐related genes displayed higher expression levels in both GLY‐injured and CTX‐injured muscles than that in non‐injured muscles (*Figure* S2J). Notably, expression levels of *Col3a1*, *Col1a1*, and *Col1a2* in GLY‐injured muscle were higher than that in the CTX‐injured muscle (*Figure* S2J), suggesting that collagen development plays a more important role in the GLY‐injured muscle at 5 DPI.

### Clustering and pseudotemporal trajectories identified transcriptional dynamics of myeloid‐derived cells

**Figure 3:**
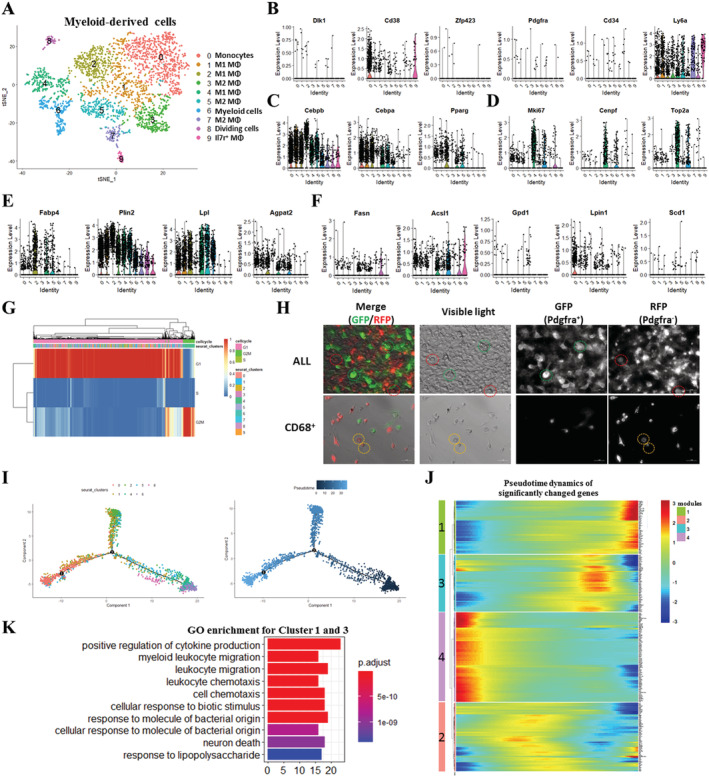
Clustering and pseudotemporal trajectories identified transcriptional dynamics of myeloid‐derived cells. (A) Graph‐based clustering of myeloid‐derived cells showing 10 subclusters. (B) Expression of adipocyte‐enriched genes (*Dlk1*, *Cd38*, *Zfp423*, *Pdgfra*, *Cd34*, and *Ly6a*). (C) Expression of adipogenic master regulators (*Cebpb*, *Cebpa*, and *Pparg*). (D) Expression of proliferation genes (*Mki67* and *Cenpf*). (E) Expression of lipid synthesis genes (*Fabp4*, *Plin2*, *Lpl*, and *Agpat2*). (F) Expression of lipid metabolism genes (*Fasn*, *Acsl1*, *Gpd1*, *Lpin1*, and *Scd1*). (G) Cell cycle analysis of myeloid‐derived cells. (H) Fluorescence and visible light micrographs of all single cells and Cd68^+^ cells isolated from GLY‐injected TA of Pdgfra^cre^/ROSA^mT/mG^ mice after adipogenic differentiation. Red circles: Pdgfra^−^ cells with lipid droplets; green circles: Pdgfra^+^ cells with lipid droplets; and yellow circles: Pdgfra^−^/Cd68^+^cells with lipid droplets. (I) Pseudotime single‐cell trajectory reconstructed by Monocle2 for seven subclusters of myeloid‐derived cells, including Mye0, Mye1, Mye2, Mye4, Mye5, Mye6, and Mye8. Pseudotime is shown coloured in a gradient from dark to light blue, and start of pseudotime is indicated. (J) Pseudotemporal heat map showing gene expression dynamics for significant marker genes. Genes (rows) were clustered into four modules, and cells (columns) were ordered according to pseudotime. (K) GO enrichment analysis of genes in Modules 1 and 3.

Heterogeneity of myeloid lineage has long been recognized and, in part, is a result of the heterogeneous cell origin and the specialization of tissue macrophages in particular microenvironments. The physiological function of these heterogeneous myeloid‐derived cells is not completely understood. We performed t‐SNE analysis on cluster myeloid‐derived cells and observed the presence of 10 juxtaposed subclusters and further identified these subclusters based on the expression levels of myeloid‐derived cells marker genes (*Cd68*, *Clec12a*, and *Acp5*), M1 macrophage (M1 MΦ; *Fabp4* and *Pf4*), M2 MΦ (*Cxcl3* and *Ccl6*), Il7r^+^ MΦ (*Il7r*), and monocytes (*Csf1r* and *Adgre1*) (*Figures* 3A and S3A). Visualization of the top 10 most variably expressed genes between subclusters showed distinct transcriptional programs of the 10 subclusters (*Figure* S3B). A few myeloid‐derived cells expressed these preadipocyte‐enriched genes (*Dlk1*, *Zfp423*, *Pdgfra*, and *Cd34*) (*Figure* 3B). Myeloid cells in subcluster Mye0 and Mye9 expressed relatively higher levels of *Cd38* and *Ly6a* (*Figure* 3B). The adipogenic differentiation regulators (*Cebpb*, *Cebpa*, and *Pparg*) were expressed in subclusters Mye2, Mye4, and Mye5 (*Figure* 3C). Subcluster Mye4 was also active for expressing the proliferation markers *Mki67*, *Cenpf*, and *Top2a* (*Figure* 3D). In addition, the lipid synthesis marker (*Fabp4*), perilipin marker (*Plin2*), lipoprotein lipase (*Lpl*), and the phospholipid biosynthesis enzyme (*Agpat2*) were mostly expressed in subclusters Mye2, Mye4, and Mye5 (*Figure* 3E). A few myeloid‐derived cells in subclusters Mye0, Mye1, Mye2, Mye4, Mye5, Mye6, and Mye8 were also expressed other lipid metabolism‐related genes (*Fasn*, *Acsl*, *Gpd1*, *Lpin1*, and *Scd1*) (*Figure* 3F). Cell cycle analysis based on mouse cell cycle markers revealed that 91.9% myeloid‐derived cells were G_1_‐stage cells (*Figures* 3G and S3C) while 7.8% myeloid‐derived cells were G_2_/M‐stage cells and most of G_2_/M‐stage cells belonged to subcluster Mye4 (*Figures* 3G and S3C). We next isolated Pdgfra^+^, Pdgfra^−^/Cd68^+^, and Pdgfra^−^/Cd68^−^ cells from GLY‐injured muscle at 5 DPI using MACS and found abundant and big lipid droplets in Pdgfra^+^ cells after adipogenic differentiation (*Figure* S3D). Notably, a few Pdgfra^−^/Cd68^+^ cells also displayed small lipid droplets after induction of adipogenesis (*Figure* S3D). We also generated *Pdgfra‐mT/mG* mice and isolated all single cells or Cd68^+^ cells from GLY‐injured muscle at 5 DPI and exposed them to induction of adipogenesis (*Figure* S3E). Consistently, lipid droplets were detected in both differentiated Pdgfra^+^ (green circles) and Pdgfra^−^ (red circles) cells (*Figure* 3H). Especially, lipid droplets were also detected in Pdgfra^−^/Cd68^+^ (orange circles) cells (*Figure* 3H). The results suggest that a few subpopulations of myeloid‐derived cells might have the potential to participate in lipid deposition in GLY‐injured muscle and displayed an active lipid metabolism, especially in subclusters Mye2, Mye4, and Mye5, which might be derived from proliferating cell in subcluster Mye4. To obtain temporal resolution of these subclusters, we employed the pseudotemporal ordering algorithm Monocle2^24^ on subclusters Mye0, Mye1, Mye2, Mye4, Mye5, Mye6, and Mye8. Ordering of cells in pseudotime arranged most of myeloid‐derived cells into a major trajectory, with three minor bifurcations and six stages (*Figures* 3I, S3F, and S3G). Cells located in the initial phase of the trajectory (start of pseudotime) corresponded to parts of subcluster Mye4, as indicated by the expression of proliferation marker genes (*Mki67* and *Cenpf*) (*Figures* 3I and S3F). The significantly affected genes among these seven subclusters along the pseudotime trajectory were assigned to four gene modules (*Figure* 3J and *Table* S5). Gene ontology analysis of up‐regulated genes in Module 1 and Module 3 indicated a positive regulation of cytokine production and myeloid leucocyte migration (*Figure* 3K). Gene ontology analysis of down‐regulated genes in Module 2 and Module 4 indicated reinforced mitotic nuclear division and ATP metabolic processes (*Figure* S3H). Furthermore, we identified the top 20 most variably expressed genes of myeloid‐derived cells cluster among non‐injured, CTX‐injured, and GLY‐injured muscles (*Figure* S4A). Several adipogenic or lipogenic genes (*Ly6a*, *Cebpb*, *Fabp4*, *Plin2*, and *Lpl*) had higher expression levels in GLY‐injured group compared with the non‐injured or CTX‐injured group (*Figure* S4B). Taken together, several subclusters of myeloid‐derived cells were involved in fat infiltration and lipid metabolism through lipid deposition or cytokine production during GLY‐induced muscle regeneration.

### Glycerol‐induced intramuscular fat infiltration changes in the overall composition of lipids in skeletal muscle

**Figure 4:**
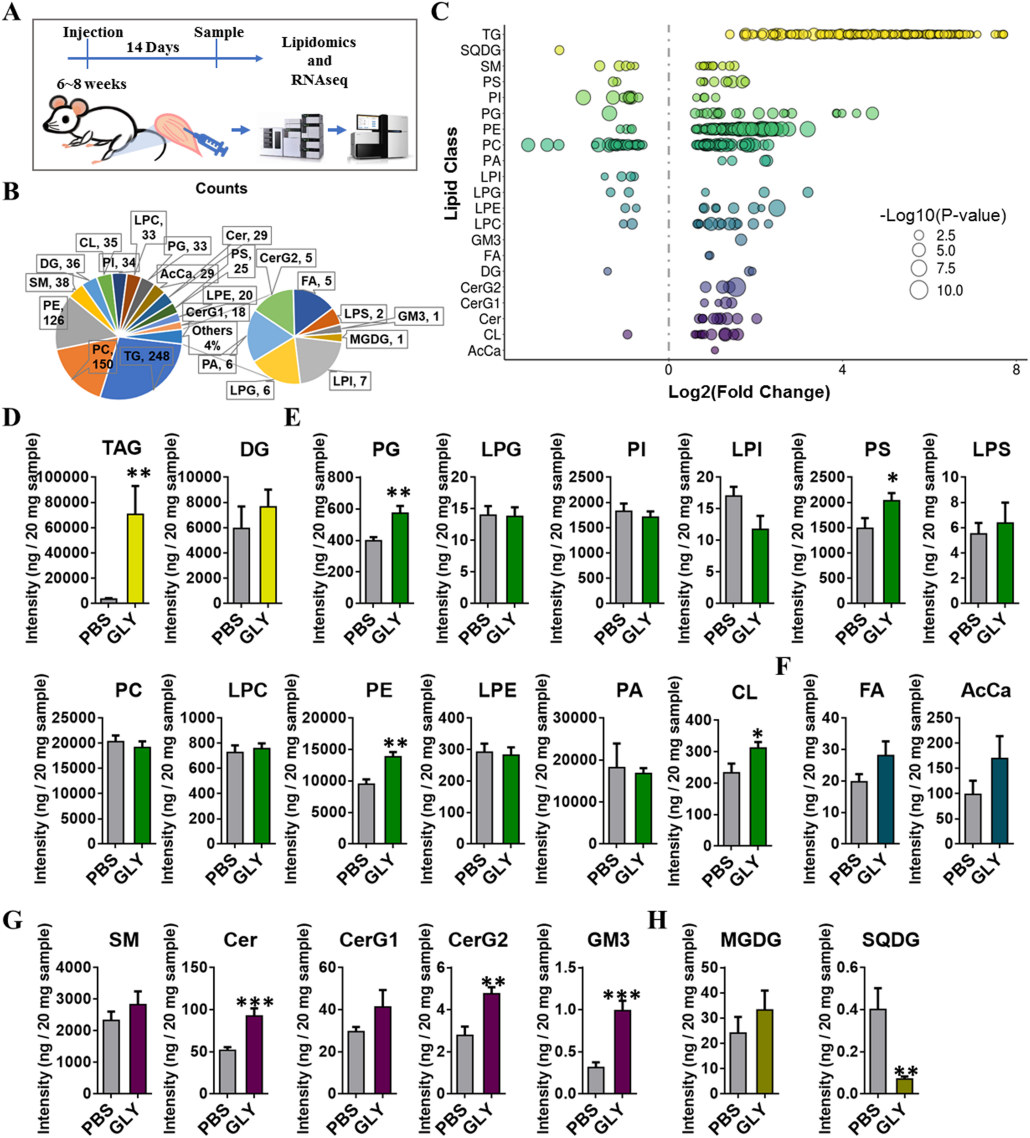
GLY‐induced IMAT infiltration changes in the overall composition of lipids in skeletal muscle. (A) Scheme of muscle preparation and lipidomic analysis of NACL‐injected or GLY‐injected TA at 14 DPI. (B) Composition of lipid classes that were considered for subsequent analysis in all of the samples detected by liquid chromatography–mass spectrometry/mass spectrometry. (C) Log_2_ fold changes in lipid species in NACL‐injected vs. GLY‐injected TA and the corresponding significance values displayed as −log_10_ (*P*‐value). Each dot represents a lipid species, and the dot size indicates significance. Only lipids with *P* < 0.05 are displayed (*n* = 8). The intensity fold change of (D) glycerolipids, (E) glycerophospholipids, (F) fatty acyls, (G) sphingolipids, and (H) saccharolipids in NACL‐injected vs. GLY‐injected TA. Data are presented as means ± standard error of the mean (*n* = 8). *P < 0.05; ***P < 0.001.

To examine lipid metabolism and lipidomic changes during fat infiltration in skeletal muscle, we conducted a mass spectrometry‐based lipidomic analysis of GLY‐injected TA muscles at 14 DPI (*Figure* 4A). As expected, GLY injection leads to much more cellular structures devoid of eosin‐positive cytoplasm, which are reminiscent of mature white adipocytes containing triglycerides (TAGs) in a large lipid droplet (*Figure* S5A). The mRNA levels of adipogenic genes (such *Adipoq*, *Scd1*, and *Ppara*) and mitochondrial genes (*Ucp1*, *Ppara*, and *Cox5a*) were strongly induced by GLY injection (*Figure* S5B). Consistently, the content of TAGs in muscle was significantly increased following GLY injection (*Figure* S5C). These results suggest that GLY injection may induce apparent IMAT accumulation. IMAT content in skeletal muscle is closely related to the whole‐body metabolism. However, the body weights and food and water intakes showed no difference between the GLY‐injected and NACL‐injected groups (*Figure* S5D). There was no change in the mass of TA, brown adipose tissue (BAT), inguinal white adipose tissue, and epididymal white adipose tissue (*Figure* S5E). The insulin sensitivity was also not affected by fat infiltration in the GLY‐injected skeletal muscle (*Figure* S5F and S5G).

Our mass spectrometry‐derived lipidomic data revealed 888 different lipid species in muscle, consisting of 248 TAGs, 150 phosphatidylcholines (PCs), 126 phosphatidylethanolamines (PEs), and other lipid classes (*Figure* 4B). We visualized all the significantly changed lipid species by using a bubble map (*Figure* 4C). Using a *P*‐value of 0.05 as cut‐off, a total of 503 species were significantly changed in the GLY‐injected muscles (*Figure* 4C). Consistent with our previous results (*Figure* S5C), the overall abundance of TAGs, which is the most abundant lipid class in muscle, was significantly increased by GLY injection (*Figure* 4D). There were significant increases in the abundance of phosphatidylglycerol (PGs), phosphatidylserines (PSs), PEs, and cardiolipins, which belong to the second most abundant lipid class, glycerophospholipids (*Figure* 4E). The abundance of fatty acids and acyl carnitines were not affected by GLY injection (*Figure* 4F). Sphingolipids, including ceramides, simple glc series 2 (CerG2), and ganglioside 3 (GM3), were also significantly increased in GLY‐injected muscle (*Figure* 4G). In contrast, we detected a significant decrease in the concentration of sulfoquinovosyl diacylglycerol, which belong to the group of saccharolipids (*Figure* 4H). These results suggest that GLY injection induced IMAT infiltration during muscle regeneration, which affected the overall composition of lipids in the injured muscle. Next, we ranked TAG lipids with VIP > 1.0 according to the *P*‐values, compared GLY‐injected with NACL‐injected groups, and examined the top 10 species individually. All the top 10 TAG lipids significantly increased to a great extent in GLY‐injected muscle (*Figure* S6A). And seven of the top 10 TAG lipids contained C18:1, six of the top 10 TAG lipids contained C18:2, and five of the top 10 TAG lipids contained C16:0 (*Figure* S6A). We analysed individual fatty acyl chain composition associated with TAG. Major fatty acyl chains, including C12:1, C18:0, C18:3, C16:1, C18:2, C16:0, and C18:1, were largely increased in GLY‐injected muscle (*Figure* S6B). Middle fatty acyl chains, including C24:2, C20:5, C22:5, C24:1, C22:4, C20:3, C20:4, C22:1, C22:6, and C20:0, were significantly increased in the GLY‐injected muscle (*Figure* S6C). Minor fatty acyl chains, including C26:1, C20:2, and C14:3 increased significantly in GLY‐injected muscle (*Figure* S6D). In addition, significant increases in the concentration of odd‐numbered fatty acyl chains, including C21:1, C11:0, C13:0, C21:0, C19:1, C17:1, C17:0, and C15:0, were found in the TAG pool of muscles with high‐IMAT contents (*Figure* S6E). Moreover, we analysed the total percentage of saturated fatty acids (SFAs), monounsaturated fatty acids (MUFAs), and polyunsaturated fatty acids (PUFAs) associated with TAG acyl chains. IMAT infiltration significantly decreased SFA and MUFA percentages, while compensatorily the muscle PUFA percentage increased (*Figure* S6F). The increased unsaturation of fatty acyl chains was consistent with an increase in mRNA levels of *Scd1* (*Figure* S4B). The MUFA/PUFA ratio was decreased in GLY‐injected muscle vs. controls (*Figure* S6G). These results suggest that GLY‐IMAT infiltration induced through GLY injection and affected fatty acyl chain composition associated with TAG in the muscle.

### Glycerol‐induced intramuscular fat infiltration affects gene expression involved in lipid metabolism

**Figure 5:**
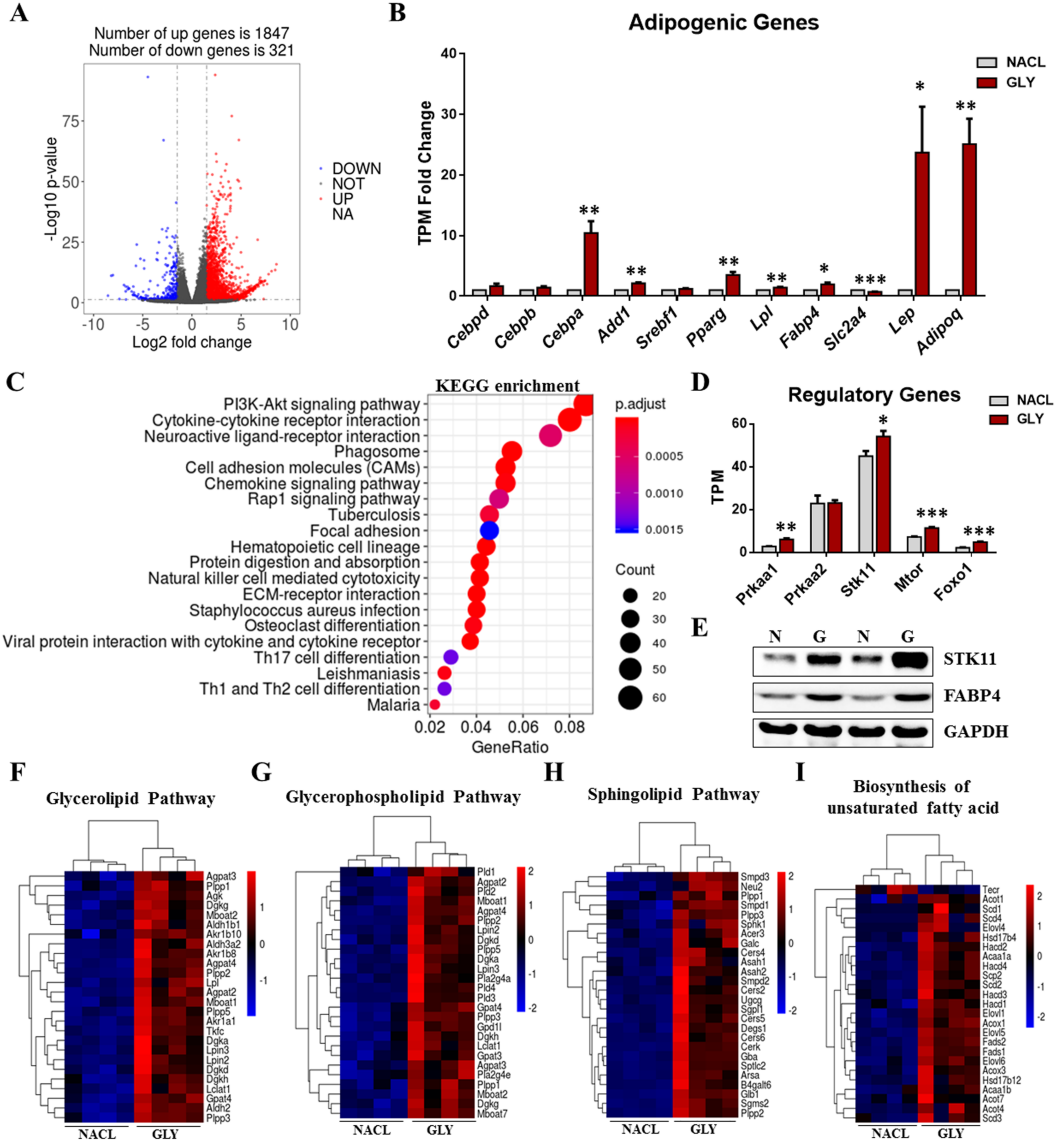
GLY‐induced IMAT infiltration affects gene expression involved in lipid metabolism. (A) Log_2_ fold changes in exons of RNA‐Seq gene bodies in NACL‐injected and GLY‐injected TA (*n* = 4) and the corresponding significance values displayed as log_10_ (*P*‐value). The transverse and vertical dotted lines indicate the cut‐off value for differential expression (*P* < 0.05 and abs (log_2_ fold changes) > 1). In total, 1847 and 321 genes were identified that had induced (red) or repressed (blue) expression levels by GLY injection. (B) TPM fold change of adipogenic genes (*Cebpd*, *Cebpb*, *Cebpa*, *Add1*, *Srebf1*, *Pparg*, *Lpl*, *Fabp4*, *Scl2a4*, *Lep*, and *Adipoq*) (*n* = 4). (C) Functional enrichment analyses using Kyoto Encyclopedia of Genes and Genomes (KEGG) pathways. The triangle size indicates significance and corresponding significance values displayed as log_10_ (*P*‐value). (D) TPM levels of lipid metabolism regulatory genes (*Prkaa1*, *Prkaa1*, *Stk11*, *Mtor*, and *Foxo1*) (*n* = 4). (E) The protein levels of STK11, FABP4, and GAPDH in NACL‐injected and GLY‐injected TA (*n* = 2). Heat map showing relative expression of (F) the glycerolipid pathway, (G) the glycerophospholipid pathway, (H) biosynthesis of unsaturated fatty acids, and (I) the sphingolipid pathway‐related genes derived from the RNA‐seq dataset.

To map transcriptional changes in skeletal muscle with IMAT infiltration, we performed RNA‐seq on GLY‐injected and NACL‐injected skeletal muscle at 14 DPI. Using a significance level of *P*‐value <0.05 and |log_2_(fold change)| > 1, we found a total of 2168 DEGs, of which 1847 and 321 genes showed increased and reduced expression, respectively (*Figure* 5A). The expression of these genes related to adipogenesis (*Cebpa*, *Add1*, *Pparg*, *Lpl*, *Fabp4*, *Lep*, and *Adipoq*) was significantly increased by GLY‐induced fat infiltration (*Figure* 5B). Notably, the expression level of *Slc2a4*, which encodes glucose transporter type 4, was down‐regulated in GLY‐injected muscle (*Figure* 5B). GO enrichment analysis of the DEGs revealed pronounced changes in the extracellular matrix (ECM), tin cell migration, and cell adhesion (*Figure* S7A). Functional enrichment analyses using the KEGG pathway^33^, ^34^ revealed a significant enrichment of ECM–receptor interaction pathway, PI3K–AKT signalling pathway, haematopoietic cell differentiation, and chemokine signalling pathways (*Figures* 5C and S7B). The expression of lipid metabolism regulatory genes (*Prkaa1*, *Stk11*, *Mtor*, and *Foxo1*), which serve downstream of the PI3K–AKT signalling pathway, also increased in high‐IMAT muscle (*Figure* 5D). Protein levels of STK11 were remarkably increased, coincident with increased FAPB4 level in GLY‐injected muscles (*Figure* 5E). Our lipidomic analysis revealed significant changes in glycerolipids, glycerophospholipids, and sphingolipids. The TPM analysis showed notable differences in genes involved in glycerolipid, glycerophospholipid, and sphingolipid metabolism between fat‐infiltrated muscles and controls (*Figure* 5F–5H). In addition, biosynthesis of unsaturated fatty acid‐related genes was also affected by high‐IMAT contents (*Figure* 5I), which is consistent with the alteration of fatty acyl chains associated with TAG. These results suggested that GLY‐induced fat infiltration simultaneously altered the expression levels of genes involved in ECM, cell migration, and cell adhesion, as well as in lipid and fatty acid metabolism pathways.

### Cold exposure altered the fatty acid composition in intramuscular fat‐infiltrated tibialis anterior

**Figure 6:**
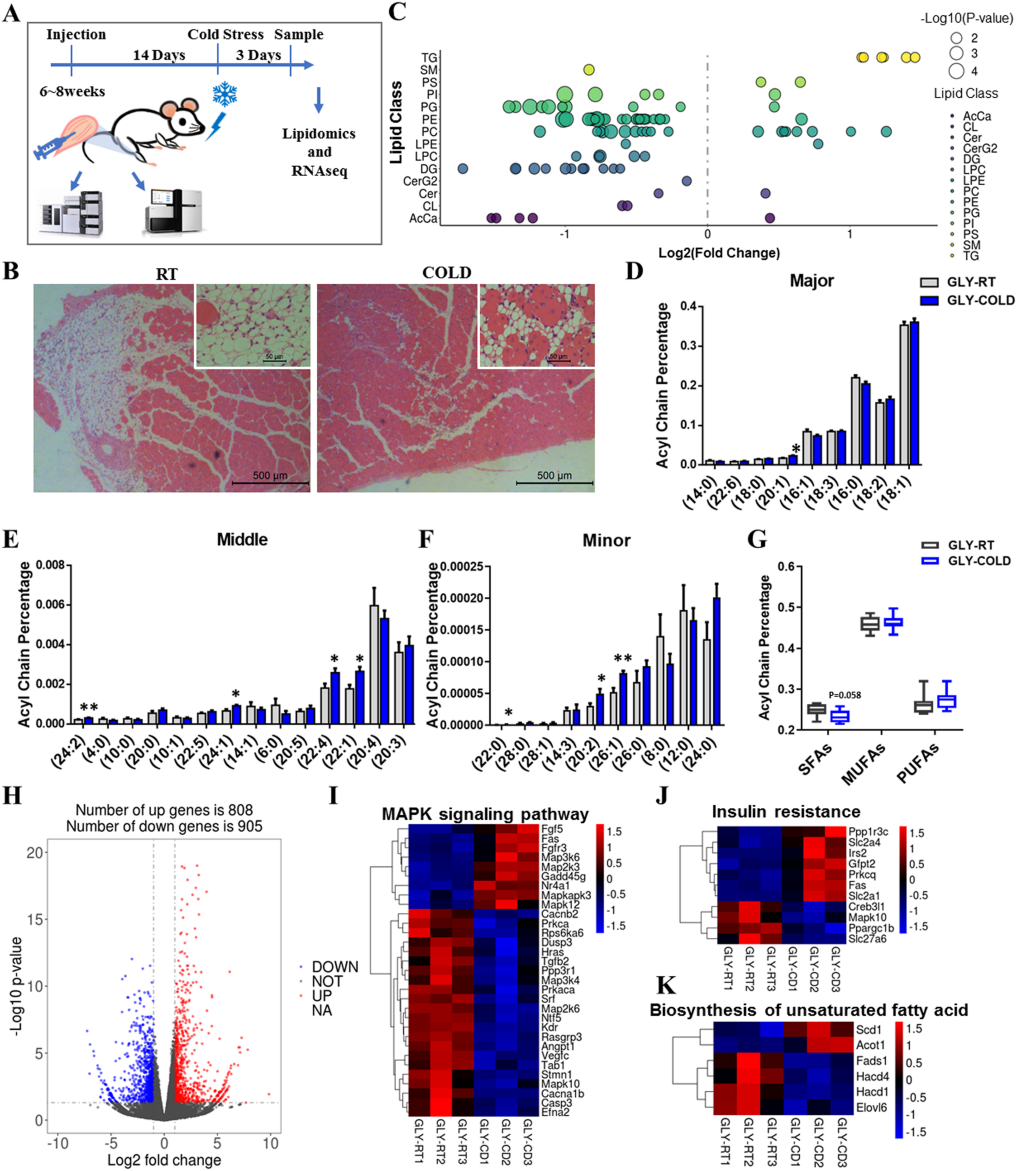
Cold exposure alters the fatty acid composition in IMAT‐infiltrated TA. (A) Scheme of muscle preparation and lipidomic analysis of GLY‐induced IMAT‐infiltrated (after 14 days) TA from cold‐exposed (3 days) and RT mice. (B) H&E staining of IMAT‐infiltrated TA from cold‐exposed and RT mice (*n* = 3). Scale bars, 100 mm. (C) Log_2_ fold changes in lipid species in IMAT‐infiltrated TA from cold‐exposed and RT mice and the corresponding *P*‐values displayed as −log_10_ (*P*‐value). Each dot represents a lipid species, and dot size indicates significance. Only lipids with *P* < 0.05 are displayed (*n* = 8). (D–F) The total intensity of individual fatty acyl chains associated with TAG (*n* = 8). (G) Percentages of SFA, MUFA, and PUFA in TAG acyl chain in IMAT‐infiltrated TA from cold‐exposed and RT mice (*n* = 8). (H) Log_2_ fold changes in exons of RNA‐seq gene bodies in IMAT‐infiltrated TA from cold‐treated and RT mice (*n* = 4) and the corresponding *P*‐values displayed as log_10_ (*P*‐value). The transverse and vertical dotted lines indicate the cut‐off values for differential expression (*P* < 0.05 and abs (log_2_ fold changes) > 1). In total, 808 and 905 genes were identified that had increased (red) or lowered (blue) expression levels due to cold exposure. (I–K) Heat map of relative expression of the MAPK signalling, insulin resistance, and unsaturated fatty acid biosynthesis pathway‐related genes from the RNA‐seq dataset of RT vs. COLD groups.

To examine the short‐term effects of cold exposure in fat‐infiltrated skeletal muscle, we maintained mice, previously injected with GLY and having IMAT infiltration at either RT (22°C) or cold conditions (4°C) for 3 days and closely examined the body and tissue weights (*Figure* 6A). We found that cold exposure resulted in body weight losses as well as losses in adipose tissue mass (BAT, inguinal white adipose tissue, and epididymal white adipose tissue) and TA (*Figure* S8A). H&E staining revealed an obvious decrease in cell number of IMAT in cold‐exposed mice compared with RT individuals (*Figure* 6B). Cold exposure increased the cross‐sectional area of the centronuclear myofibres and decreased the cross‐sectional area of IMAT (*Figure* S8B). In addition, cold exposure increased the expression of BAT marker genes (*Ucp1*, *Prdm16*, and *Ppara*) (*Figure* S7C). However, *Cidea* and *Pgc1a* significantly decreased (*Figure* S8C). Lipid and energy metabolism‐related genes (*Leptin* and *Scd1*, *Ucp2*, *Ucp3*, and *Cox5a*) also increased in cold‐exposed fat‐infiltrated skeletal muscle (*Figure* S8D and S8E). Notably, the muscle differentiation marker (*Myod1*) significantly increased after cold exposure (*Figure* S8F). Our data demonstrate that 3 day cold exposure decreased body weight and fat masses, induced the expression of BAT marker genes of GLY‐induced IMAT, affected cellular lipid and energy metabolism, and decreased adipocyte infiltration in the GLY‐induced IMAT‐infiltrated models.

Next, we determined overall changes in lipid composition of fat‐infiltrated skeletal muscle after cold exposure. Short‐term cold exposure had no significant effect on the overall composition of lipid classes (*Figure* S9A–S9E). Using a *P*‐value of 0.05 as cut‐off, a total of 98 species were significantly affected in fat‐infiltrated skeletal muscle after cold exposure, which was shown in a bubble map (*Figure* 6C). We next analysed the percentage of individual fatty acyl chains associated with TAG (*Figure* 6D–6F and S9F). Significant increases in the percentage of MUFAs (C20:1, C24:1, C22:1, and C26:1) and PUFAs (C24:2, C22:4, and C20:2) were observed even after short time in the cold exposure group (*Figure* 6D–6F). There was also a significant increase in the percentage of SFAs (C22:0). Moreover, we analysed the total percentage of SFA, MUFA, and PUFA associated with TAG acyl chains. Cold exposure decreased the percentage of SFA, without affecting MUFA or PUFA in fat‐infiltrated skeletal muscle after cold exposure (*Figure* 6G).

To explore how actually the lipidome in fat‐infiltrated skeletal muscle was affected by cold exposure in high‐IMAT muscles, we applied RNA‐seq to map the transcriptional changes and lipid metabolic pathways. Using a significance level of *P*‐value <0.05 and |log_2_(fold change)| > 1, we found a total of 1713 DEGs, of which 808 and 905 genes show increased and reduced expression, respectively (*Figure* 6H). GO enrichment analysis of the DEGs revealed pronounced changes in the ECM, microtube, and mitotic cell cycle (*Figure* S10A). Consistently, functional enrichment analyses using KEGG pathway revealed a significant enrichment of ECM–receptor interaction pathway, PI3K–AKT signalling pathway, MAPK signalling pathway, insulin resistance pathway, and cell cycle pathways (*Figure* S10B and S10C). The TPM analysis showed slight differences of genes involved in MAPK signalling and insulin resistance pathways (*Figure* 6I and 6J). Our lipidomic analysis did not reveal significant changes in glycerolipid, glycerophospholipid, and sphingolipid compositions. The TPM analysis showed small differences in genes involved in glycerolipid, glycerophospholipid, and sphingolipid metabolism, between the high‐IMAT and control groups (*Figure* S10D). In addition, a small part of the unsaturated fatty acid pathway, fatty acid biosynthesis, fatty acid degradation, and fatty acid elongation‐related genes was affected by IMAT infiltration (*Figures* 6K and S10E). However, most of the oxidative phosphorylation pathway‐related genes were decreased in the cold‐exposed fat‐infiltrated skeletal muscle (*Figure* S10F). These results suggest that cold exposure alters the fatty acid composition of lipids in GLY‐induced fat‐infiltrated skeletal muscle through directly affecting lipid metabolism pathways.

## Discussion

In this study, we generated a GLY‐induced IMAT infiltration mouse model and applied scRNA‐seq, mass spectrometry‐based lipidomics, and RNA‐seq to provide a comprehensive resource describing the molecular signatures of fat infiltration in skeletal muscle. By single‐cell transcriptomics, we constructed a cell atlas of GLY‐injured skeletal muscle and revealed the heterogeneity of fibroblast/FAPs and myeloid‐derived cells and inferred adipogenic niches of IMAT infiltration during GLY‐induced skeletal muscle regeneration. Our lipidomic results showed that GLY‐induced IMAT infiltration affected the overall composition of lipid classes and the length of acyl chains associated with TAG in skeletal muscle. RNA‐seq results indicated an important role of the extracellular matrix and immune cells in the progress of GLY‐induced skeletal muscle regeneration. Moreover, our mass spectrometry‐based lipidomics and RNA‐seq revealed the lipidomic and transcriptomic profiles of GLY‐induced fat‐infiltrated skeletal muscle after a 3‐day cold exposure. Our results further indicated that cold exposure did not affect the overall composition of lipid classes but induced significant changes in the length of acyl chains associated with TAG in GLY‐induced fat‐infiltrated skeletal muscle. RNA‐seq results indicated that MAPK signalling, insulin resistance, and lipid metabolism‐related pathways play a crucial role in response to cold exposure in GLY‐induced fat‐infiltrated skeletal muscle.

It has been reported that intramuscular injection of hypertonic solution of GLY altered myofibre permeability and disrupted the basal lamina, especially resulting in the accumulation of ectopic adipocytes, which makes it distinguishable from the other muscle injury models (such as CTX, freezing, and ischaemia reperfusion).^22^ Intramuscular GLY injection is well suitable for investigating the pathology of muscular diseases and mechanisms regulating adipogenesis in skeletal muscle.^22^ In the present study, we found that GLY (50%, 7 M/L) in TA induced severe injury at 5 DPI and vast ectopic adipocyte infiltration from 14 DPI. GLY is an important circulating metabolic fuel and exists at striking lower levels in healthy tissues compared with exogenously administered reagents.^35^, ^36^ The circulating endogenous GLY provided by the breakdown of TAG in adipose tissue can be extracted and utilized by skeletal muscle and then phosphorylated into glycerol 3‐phosphate and incorporated into specific lipids.^37^, ^38^, ^39^ Thus, the residual extraneous GLY may well participate in ensuring regeneration, adipogenesis, and lipid metabolism. However, the major pitfall of our current work is not having a control condition (non‐injured) and missing the opportunities to examine temporal dynamics of fat infiltration. Thus, it will be meaningful for explaining the alterations in adipogenesis, lipogenesis, and lipid metabolism in the GLY‐injured model to identify the location of extraneous GLY in specific cells and lipids during injury and regeneration of GLY‐injected skeletal muscles by isotope tracer methods. Future work will provide a more comprehensive understanding of temporal dynamics of fat infiltration and lipid metabolism to compare with other injury models at different stages of muscle regeneration.

The effective regenerative capacity of skeletal muscles relies on muscle satellite cells (MuSCs) and their interplay with different muscle‐resident cell types within the niche, such as FAPs,^40^ macrophages,^41^ and ECs.^42^ Recently, scRNA‐seq has been applied to studying cellular heterogeneity in this complex tissue.^32^, ^43^ Giordani *et al*. mapped eight already known muscle‐resident cell types (B cells, T cells, macrophages, neutrophils, ECs, FAPs, MuSCs, and glial cells) and two previously understudied populations (Itga7^+^/Vcam1^−^ cells and Scx^+^ cells) in normal adult mouse muscles, using a combined approach of single‐cell RNA sequencing and mass cytometry.^32^ Dell'Orso *et al*. performed scRNA‐seq on hindlimb skeletal muscles of 3‐month‐old C56BL/6J mice and identified nine cell clusters including FAPs, tenocyte‐like cells, smooth muscle cells, ECs, adaptive and innate immunity cells (B cells, T cells, macrophages, and monocytes), and a small cluster expressing genes present in mature skeletal muscle (e.g. creatine kinase muscle and Ckm).^43^ In this study, we applied scRNA‐seq and characterized the cellular diversity of GLY‐injured skeletal muscles with nine major identified cell types, including myeloid‐derived cells, fibroblast/FAPs, NK cells, T lymphocytes, neutrophils, MuSCs, APCs, B lymphocytes, and ECs (*Figure* 1). In a normal muscle, scRNA‐seq analysis revealed that FAPs and ECs accounted for most of the cell populations and there were few macrophage/monocytes and neutrophils.^32^ Previous studies reported that immune cells are relatively scarce in normal skeletal muscles, while, they can be present in regenerative muscles at concentrations that exceed 100 000 inflammatory cells/mm^3^ of muscle tissue.^44^ Consistently, we found that myeloid‐derived cells (including macrophages, monocytes, and myeloid cells) became the largest cluster in GLY‐injured muscle. Consequently, percentages of other cells (e.g. ECs and MuSCs) seem to decrease in GLY‐injured muscle compared with those in normal muscles. Even pericytes were absent in major cell clusters of GLY‐injured muscle due to their low proportion. Despite of some canonical markers, the expression of other markers was not restricted to specific ‘islands’. For example, the markers for satellite cells (*Myod1*, *Myf5*, *Pax7*, or *Myog*) were not among the top 20 markers of the MuSCs cluster, because of the low mean expression levels in the MuSCs cluster. Thus, it was difficult to precisely identify all clusters obtained by unsupervised clustering of the quality‐controlled cells in skeletal muscle. Moreover, the lack of common standards to identify cell types leads to differently determined cell types in different studies. We identified natural killer cells and myofibroblasts, which were not discussed in previous scRNA‐seq studies but were, when referring to lost glial cells, Itga7^+^/Vcam1^−^ cells and Scx^+^ cells, tenocyte‐like cells, and smooth muscle cells, identified by Giordani *et al*.^32^ and Dell'Orso *et al*.^43^ Nonetheless, we could still map few individuals expressing makers of these unidentified cell types in GLY‐injured muscles. To understand the characteristics and function of these scarce cell types, future scRNA‐seq studies will have to be performed on the specific cell types purified and enriched by fluorescence‐activated cell sorting based on their makers.

Consistent with the indispensable role of FAPs on fibrosis and fat infiltration during muscle regeneration, scRNA‐seq analysis and further clustering of fibroblast/FAPs revealed that myofibroblasts expressing *Pdgfra* were largely increased in injured muscles and half of the myofibroblasts were proliferative.^45^, ^46^ Based on the pseudotemporal trajectories of fibroblast/FAPs, GO analysis revealed that the extracellular matrix and the cytokine pathways, but not adipogenesis pathways, were activated in fibroblast/FAPs at 14 DPI. Joe *et al*. reported that FAPs proliferate more quickly than MuSCs during the first 72 h after injury and produce soluble factors that stimulate MuSCs to facilitate myogenesis.^40^ The comparison of scRNA‐seq datasets of FAPs from non‐injured, CTX‐injured, and GLY‐injured muscles indicated higher expression levels of collagen and extracellular matrix‐related genes in FAPs from the GLY‐injured group. *In vitro* single‐cell culture experiments also show that not all pdgfra^+^ cells isolated from 5 day GLY‐injected muscle had the capacity to differentiate into mature adipocytes. The fate of FAPs is largely dependent on the muscle environment.^45^ Thus, fibroblast/FAPs might thus play a role in fibrosis and the production of cytokines at the early stage of GLY‐injected muscle regeneration due to the disrupted basal lamina in this model, and FAPs may participate in adipogenesis and fat accumulation at a later time point. The precise role of fibroblast/FAPs in muscle regeneration during different stages and under different conditions requires further experimental work.

In this study, the heterogeneous muscle tissue myeloid‐derived cells were subdivided into 10 subclusters, and a portion of these myeloid‐derived cell subclusters expressed adipocyte‐enriched genes (*Dlk1*, *Cd38*, *Zfp423*, and *Cd34*) and adipogenic regulators (*Cebpa* and *Pparg*). Consistent to our findings, pericytes^47^ and myeloid lineage cells^48^ can also differentiate into adipocytes in skeletal muscle. Considering the cell fusion events in myeloid lineage cells, Guerrero‐Juarez *et al*. used bone marrow transplantation and Cre recombinase‐based lineage tracing experiments and confirmed that myeloid lineage cells could give rise to rare regenerated adipocytes in murine skin wounds.^49^ We also detected lipid droplets in Pdgfra^−^/Cd68^+^ cells upon adipogenic induction using MACS and Cre recombinase‐based lineage tracing experiments and inferred that a subpopulation of myeloid‐derived cells might convert into *de novo* adipocytes around an injured dot in skeletal muscle. It has been proposed that adipose tissue macrophages can exist in a metabolically activated state with high intracellular lipid accumulation and activated lysosomal‐dependent lipid metabolism.^50^, ^51^ Subclusters of myeloid‐derived cells in GLY‐injured skeletal muscle expressing high levels of lipid synthesis genes (*Plin2*, *Agpat2*, *Fasn*, *Acsl1*, and *Lpin1*) might also exist in a metabolically activated state with high levels of intracellular lipids. Moreover, the local proliferation of myeloid‐derived cells subclusters expressing high levels of proliferative markers (*Mki67* and *Cenpf*) may account for the increased number of tissue macrophages, together with the recruitment of peripheral cells.^52^ However, the fate of these proliferative myeloid‐derived cells remains unclear. More lineage tracing and functional studies will be required to determine which myeloid‐derived cell population preferentially contributes to immune cell infiltration or fat deposition in skeletal muscle.

Previous studies have verified that adipose tissue had much more TAGs than skeletal muscle but a lower concentration of glycerophospholipids (GPs), including PGs, PSs, PEs, and PCs.^53^ An analysis between the two types of skeletal muscle (gluteus and soleus) indicated that soleus, which is characterized as slow‐twitch fibre‐predominant containing more oxidative‐type fibres,^54^ contained more PGs, PSs, PEs, PCs, and GMs than gluteus.^53^, ^55^ In our present study, the contents of total TAGs, PGs, PSs, PEs, cardiolipins, Cers, CerG1s, and GM3s were significantly increased in GLY‐injected muscles while the contents of total SQDGs were significantly decreased. The total content of PCs had no difference between high IMAT and control. However, many lipid species in PCs were significantly affected by GLY injection in muscle. About one‐half of these PC lipid species were significantly increased, and the others were significantly decreased. Although the damaged areas had been totally replaced by ectopic adipose tissue at 14 DPI, GO enrichment and KEGG enrichment analysis revealed a significant enrichment of the ECM–receptor interaction pathway, immune cell differentiation pathway, and the PI3K–AKT signalling pathway. As we used the whole injured TA, containing undamaged muscle, regenerative muscle, and ectopic adipose and infiltrated immune cells, our lipidomic and transcriptomic results may be erroneous in this respect and may reflect general changes in lipid composition and metabolism in GLY‐injured muscles. The presence of adipose and conjunctive tissues also may have led to some artefacts in the lipid compositional analyses in muscular dystrophy.^56^ Future work should reveal the functional significance of lipid metabolism located in specific tissues or cells in muscle.

As lipids are the most important compounds of cellular structure, many groups have tried to characterize biochemical lipids composition in skeletal muscles during degenerative and regenerative processes.^56^ Previous studies reported that TAG was significantly elevated in skeletal muscle of DMD, bone mineral density, and limb‐girdle muscular dystrophy type 2B patients as compared with normal/control individuals.^57^ Consistently, we found that lipid classes were remodelled selectively using untargeted lipidomics after GLY‐induced muscle regeneration, especially in terms of TAG contents. Linoleic acid was significantly reduced in muscle tissues of DMD, bone mineral density, facioscapulohumeral muscular dystrophy, and limb‐girdle muscular dystrophy type 2B.^57^ At the same time, we found that the content of C18:2 associated with TAGs was significantly increased. The unsaturation index in the lipids showed a decreased trend in gastrocnemius muscle from 6‐week‐old MDX mice but increases more quickly in 24‐week‐old MDX mice vs. controls.^58^ We found that ectopic adipose tissue induced by GLY injection significantly decreased the percentages of SFAs and MUFAs, while it significantly increased PUFAs in muscle. Consistently, qPCR analysis revealed an increased mRNA expression of *Scd1* in GLY‐injected muscle. The RNA‐seq results also indicated that a large proportion of genes were involved in the biosynthesis of unsaturated fatty acids. We also observed a significant increase of DHA (C22:6)‐TAGs and EPA (C20:5)‐TAGs in high‐IMAT muscles, which may protect against multiple metabolic and neurological disorders.^59^ As IMAT content plays a key role in various quality traits of meat, many studies have focused on nutritional manipulation and genetic strategies to increase IMAT content in muscle. Our results may also provide a strategy to increase IMAT content, dependent on lipid deposition during impaired muscle regeneration induced by GLY injection. We also assessed lipid profiles of the muscles high in ectopic adipose tissue and observed that ectopic adipose tissue increased the unsaturation of lipids stored in the muscle, which is consistently healthier for human consumption. The GLY‐injured model with large‐scale ectopic adipose infiltration corresponds more closely to severe myopathy or the old mdx mice model, suggesting that these lipid changes are related to the state of muscle degeneration. More researches based on different stages of GLY‐injured skeletal muscle regeneration are needed to investigate the lipid metabolism alterations during the pathological process of muscle disease and to find potential novel target lipid species as therapy.

Acute cold exposure and cold acclimation in rodents have been reported to markedly regulate substrate utilization, glucose uptake, muscle fibre type, and fat metabolism.^60^ In this present study, we found that short‐term cold exposure induced ‘browning’ of the ectopic adipose tissue in muscle. However, the overall concentrations of lipid classes in GLY‐injected muscle were only affected marginally by cold exposure. The alteration patterns of fatty acyl chains associated with TAGs in GLY‐injected muscles resembled BAT more than white adipose tissue.^20^, ^21^ Marcher *et al*. detected a robust increase in the levels of the C18:0, C20:0, C22:0, and C20:2 acyl chains associated with TAG in BAT upon cold exposure.^20^ In GLY‐injected skeletal muscle, the levels of C22:0 and C20:2 acyl chains associated with TAG were also significantly increased by cold exposure, and the levels of C18:0 and C20:0 showed an increased trend. On the other hand, ECM–receptor pathways, immune cell function pathways, and several cell signalling pathways but not lipid metabolism pathways were enriched in the observed transcriptomic changes, suggesting that cold exposure may have also influenced the regeneration of GLY‐injured muscle. A longer convalescence period before cold exposure may provide further insights into the effects on lipid metabolism in GLY‐injured muscle. Compared with adipose tissue, skeletal muscle, especially regenerative muscle, appears more complex and heterogeneous and may induce a more multifunctional response to cold exposure. Thus, the effects of cold exposure on different stages during muscle regeneration and on different units in injured muscle require more extensive future studies.

In conclusion, we provide a comprehensive resource describing the cell dynamics and lipidomic and transcriptomic profiles of fat deposition in skeletal muscle. Our findings could open a new avenue to understand the molecular signatures of fat infiltration in skeletal muscle, which may become useful for developing therapies for fat infiltration‐related muscle diseases.

## Funding

The project was partially supported by the National Key R&D Program of China (2018YFA0800403), the National Natural Science Foundation of China (31722053 and 31672427), the Natural Science Foundation of Zhejiang Province (LR17C170001), and the ‘Hundred Talents Program’ funding from Zhejiang University to T.S.

## Author contributions

T.S. and Z.X. designed the experiments and wrote the paper. Z.X., W.Y., Y.Z., and Q.N. conducted the experiments. Z.X. and T.S. analysed the data. W.C. conducted the experiments during the revision. T.G.V. and Y.W. assisted in the interpretation and revising of the article. All authors have read and approved the final manuscript.

## Conflict of interest

None declared.

## Supporting information


**Figure S1.** scRNA‐seq identified distinct cell populations in GLY‐injured skeletal muscle. (A) H&E staining of control and GLY‐injected TA sections on DPI 5 (*n* = 3). Scale bars, 500 μm. (B) The results obtained from Cell Ranger analyses. (C) Quality control for scRNA‐seq datasets. (D) Cell number of scRNA‐seq datasets before or after filter. (E) Cell numbers of individual cell clusters. (F) Expression of representative genes in distinct cell clusters, including macrophage/monocytes (*Cd68*), myofibroblasts (*Myl9*), natural killer cells (*Gzma*), fibroblast/FAPs (*Pdgfra*), T lymphocytes (*Cd28*), neutrophils (*Cd14*), skeletal muscle stem cells (*Myod1*), CD4/CD8 T cells (*Ccr7*), B lymphocytes (*Cd19*) and endothelial cells (*Pecam1*).
**Figure S2.** Clustering and pseudotemporal trajectories identify transcriptional dynamics of Fibroblast/FAPs. (A) Cell cycle analysis of myeloid‐derived cells. (B) Cell numbers and percent of fibroblast/FAPs in G1, S and G2/M phase. (C) Expression of mature adipocyte markers (*Fabp4*, *Adipoq*, *Plin1*, *Lep*, *Slc2a4*). (D) Pseudotime single cell trajectory reconstructed by Monocle2 for fibroblasts/FAPs. (E) Pseudotime single cell trajectorys for each subclusters of fibroblast/FAPs. (F) Expression of house‐keeping genes (*Vcp*, *Psmb2*, *Psmb4*) in new merged dataset of un‐injured and GLY‐injured skeletal muscle. (G) Expression of house‐keeping genes (*Vcp*, *Psmb2*, *Psmb4*) in new merged dataset of non‐injured, CTX‐injured and GLY‐injured skeletal muscle. (H) The t‐SNE plot of merged isolated single cells form non‐injured, CTX‐injured and GLY‐injured skeletal muscle. (I) Graph‐based clustering and cell types indentation of merged isolated single cells form non‐injured, CTX‐injured and GLY‐injured skeletal muscle. (J) Heatmap of top 20 significant genes between non‐injured, CTX‐injured and GLY‐injured fibroblast/FAPs.
**Figure S3.** Clustering and pseudo temporal trajectories identified transcriptional dynamics of myeloid‐derived cells. (A) Expression of myeloid‐derived cells marker genes (*Cd68*, *Clec12a*, *Acp5*), M1 macrophage (M1 MΦ; *Fabp4*, *Pf4*), M2 MΦ (*Cxcl3*, *Ccl6*), Il7r^+^ MΦ (*Il7r*), monocytes (*Csf1r*, *Adgre1*). (B) Heatmap representing the top 10 most differentially expressed genes between macrophage/monocytes sub‐clusters identified. Colours and numbers correspond to the cell clusters shown in B. (C) Cell numbers and percent of macrophage/monocytes in G1, S and G2/M phase. (D) Fluorescence light micrographs of Pdgfra^+^, Pdgfra^−^/Cd68^−^ and Pdgfra^−^/Cd68^−^ cells isolated from GLY‐injected TA of wild‐type mice after adipogenic differentiation incubating with Bodipy (green; lipid droplets) and Hoechst (blue; nucleus). (E) Scheme of GLY‐injected TA from *Pdgfra‐mT/mG* mice preparation, single cell isolation at 5 DPI and adipogenic differentiation for 5 days. (F) Pseudotime single cell trajectorys for each subclusters of fibroblasts/FAPs. (G) Pseudotime single cell trajectory is coloured by states. (H) GO enrichment analysis of genes in modules 2 and 4.
**Figure S4.** Comparison of myeloid‐derived cells between the non‐injured, CTX‐injured and GLY‐injured group. (A) Heatmap showing the top 20 significant genes between non‐injured, CTX‐injured and GLY‐injured myeloid‐derived cells. (B) Expression of adipocyte‐enriched genes (*Dlk1*, *Cd38*, *Zfp423, Pdgfra*, *Cd34*, and *Ly6a*), adipogenic master regulators (*Cebpb*, *Cebpa* and *Pparg*), lipid synthesis genes (*Adipoq*, *Fabp4*, *Plin2*, *Lpl*, *Agpat2*), lipid metabolism genes (*Fasn*, *Acsl1*, *Gpd1*, *Lpin1*, *Scd1*) in the non‐injured, CTX‐injured and GLY‐injured group.
**Figure S5.** GLY‐induced IMAT infiltration affects gene expression involved in lipid metabolism. (A) H&E staining of NACL‐ and GLY‐injected TA sections (*n* = 3). Scale bars, 100 mm. (B) mRNA expression fold change of BAT‐selective and adipocyte metabolism and muscle development related genes in NACL‐ versus GLY‐injected TA (*n* = 5). (C) TAG content of NACL‐ and GLY‐ injected TA (*n* = 6). (D) Body weight gain (*n* = 11), food intake (*n* = 4) and water intake (n = 4) of NACL‐ and GLY‐injected mice. (E) Effect of GLY‐injection in TA on mass of TA, BAT, iWAT and EWAT (n = 11). (F) GTT test of NACL‐ and GLY‐injected mice (*n* = 7). (G) ITT test of NACL‐ and GLY‐ injected mice (*n* = 6). Error bars represent SEM. * *P* < 0.05, ** *P* < 0.01, *** *P* < 0.001, two‐tailed Students t‐test.
**Figure S6.** GLY‐induced IMAT infiltration regulates the composition of fatty‐acyl chains associated with TAGs. (A) The top 10 TAGs according to the P‐Value, detected in NACL‐ and GLY‐ injected TA (*n* = 8). (B‐E) The total intensity of individual fatty‐acyl chains associated with TAGs in NACL‐ and GLY‐injected TA (*n* = 8). ODD, odd‐numbered fatty acyls. (F) Percentages of SFA, MUFA and PUFA in TAG acyl chain in NACL‐ and GLY‐injected TA (n = 8). SAF, saturated fatty acyls; MUFA, monounsaturated fatty acyls; PUFA, polyunsaturated fatty acyls containing two or three to six double bonds. (G) Total MUFA to total PUFA ratio in TAG acyl chain. Error bars represent SEM.* *P* < 0.05, ** *P* < 0.01, *** *P* < 0.001, two‐tailed Students t‐test.
**Figure S7.** GLY‐induced IMAT infiltration affects transcriptomic profiles (A) Gene Ontology (GO) enrichment analysis of significant genes in NACL‐ versus GLY‐injected TA. The triangle size indicates significance and corresponding significance values displayed as log10 (P‐value). (B) The correlation of Top 20 KEGG enrichment pathways.
**Figure S8.** Effect of cold exposure on tissue weights and lipid metabolism in GLY‐induced IMAT infiltrated TA. (A) Effect of cold exposure in GLY‐injected TA on body weight gain and mass of BAT, iWAT, EWAT and TA (*n* = 8). (B) Cross‐section area percentages of myotubes and fat in GLY‐injected TA under RT and COLD treatment. (C‐F) mRNA levels of BAT‐selective (C), adipogenesis (D), mitochondria metabolism (E) and muscle development (F) related genes in GLY‐injected TA from cold‐treated and RT mice (*n* = 5). Error bars represent SEM. * *P* < 0.05, ** *P* < 0.01, *** *P* < 0.001, two‐tailed Students t‐test.
**Figure S9.** Effect of cold exposure on overall lipid classes composition in IMAT infiltrated TA. (A‐E) The change of glycerolipids (A), glycerophospholipids (B), fatty acyls (C), sphingolipids (D) and saccharolipids (E). (F) The total intensity of individual ODD Fatty‐acyl chains associated with TAG (*n* = 8). ODD, odd‐numbered fatty acyls. Data are presented as means + SEM (n = 8). * *P* < 0.05, *** *P* < 0.001.
**Figure S10.** Cold exposure alters gene expression involved in lipid metabolism in IMAT infiltrated TA. (A) Gene Ontology (GO) enrichment analysis of significant genes in GLY‐injected TA form cold‐exposed versus RT mice. The triangle size indicates significance and corresponding significance values displayed as log10 (P‐value). (B) Functional enrichment analyses were generated using the Kyoto Encyclopedia of Genes and Genomes (KEGG). The triangle size indicates significance and corresponding significance values displayed as log10 (P‐value). (C) The correlation of Top 20 KEGG enrichment pathways. (D‐F) Heatmap showing relative expression of lipid metabolism related pathways (glycerolipid pathway, glycerophospholipid pathway, sphingolipid pathway) (D), fatty acid metabolism pathways (fatty acid biosynthesis, fatty acid depletion, fatty acid elongation) (E) and oxidative phosphorylation pathway (F) related genes from the RNA‐seq dataset of cold‐exposed versus RT groups. Only genes with *P* < 0.05 are displayed.

**Table S1** Supporting Information

**Table S2** Supporting Information

**Table S3** Supporting Information

**Table S4** Supporting Information

**Table S5** Supporting Information
