# Supplementary material for: Single‐cell RNA sequencing and lipidomics reveal cell and lipid dynamics of fat infiltration in skeletal muscle
Source: J Cachexia Sarcopenia Muscle. 2020 Nov 27;12(1):109–29. doi: 10.1002/jcsm.12643 (PMC7890272; doi:10.1002/jcsm.12643)

**Supplementary Figures**

**Figure S1. scRNA-seq identified distinct cell populations in GLY-injured skeletal muscle. (A)** H&E staining of control and GLY-injected TA sections on DPI 5 (n=3). Scale bars, 500 μm. **(B)** The results obtained from Cell Ranger analyses. **(C)** Quality control for scRNA-seq datasets. **(D)** Cell number of scRNA-seq datasets before or after filter. **(E)** Cell numbers of individual cell clusters. **(F)** Expression of representative genes in distinct cell clusters, including macrophage/monocytes (*Cd68*), myofibroblasts(*Myl9*), natural killer cells (*Gzma*), fibroblast/FAPs (*Pdgfra*), T lymphocytes (*Cd28*), neutrophils (*Cd14*), skeletal muscle stem cells (*Myod1*), CD4/CD8 T cells (*Ccr7*), B lymphocytes (*Cd19*) and endothelial cells (*Pecam1*).

**Figure S2. Clustering and pseudotemporal trajectories identify transcriptional dynamics of Fibroblast/FAPs.** (**A**) Cell cycle analysis of myeloid-derived cells. **(B)** Cell numbers and percent of fibroblast/FAPs in G1, S and G2/M phase. (**C**) Expression of mature adipocyte markers (*Fabp4*, *Adipoq*, *Plin1*, *Lep*, *Slc2a4*). **(D)** Pseudotime single cell trajectory reconstructed by Monocle2 for fibroblasts/FAPs. **(E)** Pseudotime single cell trajectorys for each subclusters of fibroblast/FAPs. **(F)** Expression of house-keeping genes (*Vcp*, *Psmb2*, *Psmb4*) in new merged dataset of un-injured and GLY-injured skeletal muscle. **(G)** Expression of house-keeping genes (*Vcp*, *Psmb2*, *Psmb4*) in new merged dataset of non-injured, CTX-injured and GLY-injured skeletal muscle. **(H)** The t-SNE plot of merged isolated single cells form non-injured, CTX-injured and GLY-injured skeletal muscle. **(I)** Graph-based clustering and cell types indentation of merged isolated single cells form non-injured, CTX-injured and GLY-injured skeletal muscle. **(J)** Heatmap of top 20 significant genes between non-injured, CTX-injured and GLY-injured fibroblast/FAPs.

**Figure S3. Clustering and pseudo temporal trajectories identified transcriptional dynamics of myeloid-derived cells.** (**A**) Expression of myeloid-derived cells marker genes (*Cd68*, *Clec12a*, *Acp5*), M1 macrophage (M1 MΦ; *Fabp4*, *Pf4*), M2 MΦ (*Cxcl3*, *Ccl6*), Il7r^+^ MΦ (*Il7r*), monocytes (*Csf1r*, *Adgre1*). (**B**) Heatmap representing the top 10 most differentially expressed genes between macrophage/monocytes sub-clusters identified. Colors and numbers correspond to the cell clusters shown in B. **(C)** Cell numbers and percent of macrophage/monocytes in G1, S and G2/M phase. **(D)** Fluorescence light micrographs of Pdgfra^+^, Pdgfra^-^/Cd68^-^ and Pdgfra^-^/Cd68^-^ cells isolated from GLY-injected TA of wild-type mice after adipogenic differentiation incubating with Bodipy (green; lipid droplets) and Hoechst (blue; nucleus). **(E)** Scheme of GLY-injected TA from *Pdgfra-mT/mG* mice preparation, single cell isolation at 5 DPI and adipogenic differentiation for 5 days. **(F)** Pseudotime single cell trajectorys for each subclusters of fibroblasts/FAPs. **(G)** Pseudotime single cell trajectory is colored by states. **(H)** GO enrichment analysis of genes in modules 2 and 4.

**Figure S4. Comparison of myeloid-derived cells between the non-injured, CTX-injured and GLY-injured group. (A) Heatmap showing the top 20 significant genes between non-injured, CTX-injured and GLY-injured myeloid-derived cells. (B) Expression of adipocyte-enriched genes (*Dlk1*, *Cd38*, *Zfp423,* *Pdgfra*, *Cd34*, and *Ly6a*), adipogenic master regulators (*Cebpb*, *Cebpa* and *Pparg*), lipid synthesis genes (*Adipoq*, *Fabp4*, *Plin2*, *Lpl*, *Agpat2*), lipid metabolism genes (*Fasn*, *Acsl1*, *Gpd1*, *Lpin1*, *Scd1*) in the non-injured, CTX-injured and GLY-injured group.**

**Figure S5.** **GLY-induced** **IMAT infiltration affects gene expression involved in lipid metabolism.** (**A**) H&E staining of NACL- and GLY-injected TA sections (n=3). Scale bars, 100 mm. (**B**) mRNA expression fold change of BAT-selective and adipocyte metabolism and muscle development related genes in NACL- versus GLY-injected TA (n=5). (**C**) TAG content of NACL- and GLY- injected TA (n=6). (**D**) Body weight gain (n=11), food intake (n=4) and water intake (n=4) of NACL- and GLY-injected mice. (**E**) Effect of GLY-injection in TA on mass of TA, BAT, iWAT and EWAT (n=11). (**F**) GTT test of NACL- and GLY-injected mice (n=7). (**G**) ITT test of NACL- and GLY- injected mice (n=6). Error bars represent SEM. * P<0.05, ** P<0.01, *** P<0.001, two-tailed Student’s t-test.

**Figure S6. GLY-induced IMAT infiltration regulates the composition of fatty-acyl chains associated with TAGs.** (**A**) The top 10 TAGs according to the P-Value, detected in NACL- and GLY- injected TA (n=8). (**B-E**) The total intensity of individual fatty-acyl chains associated with TAGs in NACL- and GLY-injected TA (n=8). ODD, odd-numbered fatty acyls. (**F**) Percentages of SFA, MUFA and PUFA in TAG acyl chain in NACL- and GLY-injected TA (n=8). SAF, saturated fatty acyls; MUFA, monounsaturated fatty acyls; PUFA, polyunsaturated fatty acyls containing two or three to six double bonds. (**G**) Total MUFA to total PUFA ratio in TAG acyl chain. Error bars represent SEM.* P<0.05, ** P<0.01, *** P<0.001, two-tailed Student’s t-test.

**Figure S7. GLY-induced IMAT infiltration affects transcriptomic profiles** (**A**) Gene Ontology (GO) enrichment analysis of significant genes in NACL- versus GLY-injected TA. The triangle size indicates significance and corresponding significance values displayed as log10 (P-value). (**B**) The correlation of Top 20 KEGG enrichment pathways.

**Figure S8. Effect of cold exposure on tissue weights and lipid metabolism in GLY-induced IMAT infiltrated TA.** (**A**) Effect of cold exposure in GLY-injected TA on body weight gain and mass of BAT, iWAT, EWAT and TA (n=8). **(B)** Cross-section area percentages of myotubes and fat in GLY-injected TA under RT and COLD treatment. (**C-F**) mRNA levels of BAT-selective (**C**), adipogenesis (**D**), mitochondria metabolism (**E**) and muscle development (**F**) related genes in GLY-injected TA from cold-treated and RT mice (n=5). Error bars represent SEM. * P<0.05, ** P<0.01, *** P<0.001, two-tailed Student’s t-test.

**Figure S9. Effect of cold exposure on overall lipid classes’ composition in IMAT infiltrated TA.** **(A-E)** The change of glycerolipids (**A**), glycerophospholipids (**B**), fatty acyls (**C**), sphingolipids (**D**) and saccharolipids (**E**). (**F**) The total intensity of individual ODD Fatty-acyl chains associated with TAG (n=8). ODD, odd-numbered fatty acyls. Data are presented as means + SEM (n = 8). * P < 0.05, *** P < 0.001.

**Figure S10. Cold exposure alters gene expression involved in lipid metabolism in IMAT infiltrated TA.** (**A**) Gene Ontology (GO) enrichment analysis of significant genes in GLY-injected TA form cold-exposed versus RT mice. The triangle size indicates significance and corresponding significance values displayed as log10 (P-value). (**B**) Functional enrichment analyses were generated using the Kyoto Encyclopedia of Genes and Genomes (KEGG). The triangle size indicates significance and corresponding significance values displayed as log10 (P-value). (**C**) The correlation of Top 20 KEGG enrichment pathways. **(D-F) Heatmap showing relative expression of lipid metabolism related pathways (glycerolipid pathway, glycerophospholipid pathway, sphingolipid pathway) (D), fatty acid metabolism pathways (fatty acid biosynthesis, fatty acid depletion, fatty acid elongation) (E) and oxidative phosphorylation pathway (F)** related genes from the RNA-seq dataset of cold-exposed versus RT groups. Only genes with P < 0.05 are displayed.

**Figure S1**

**
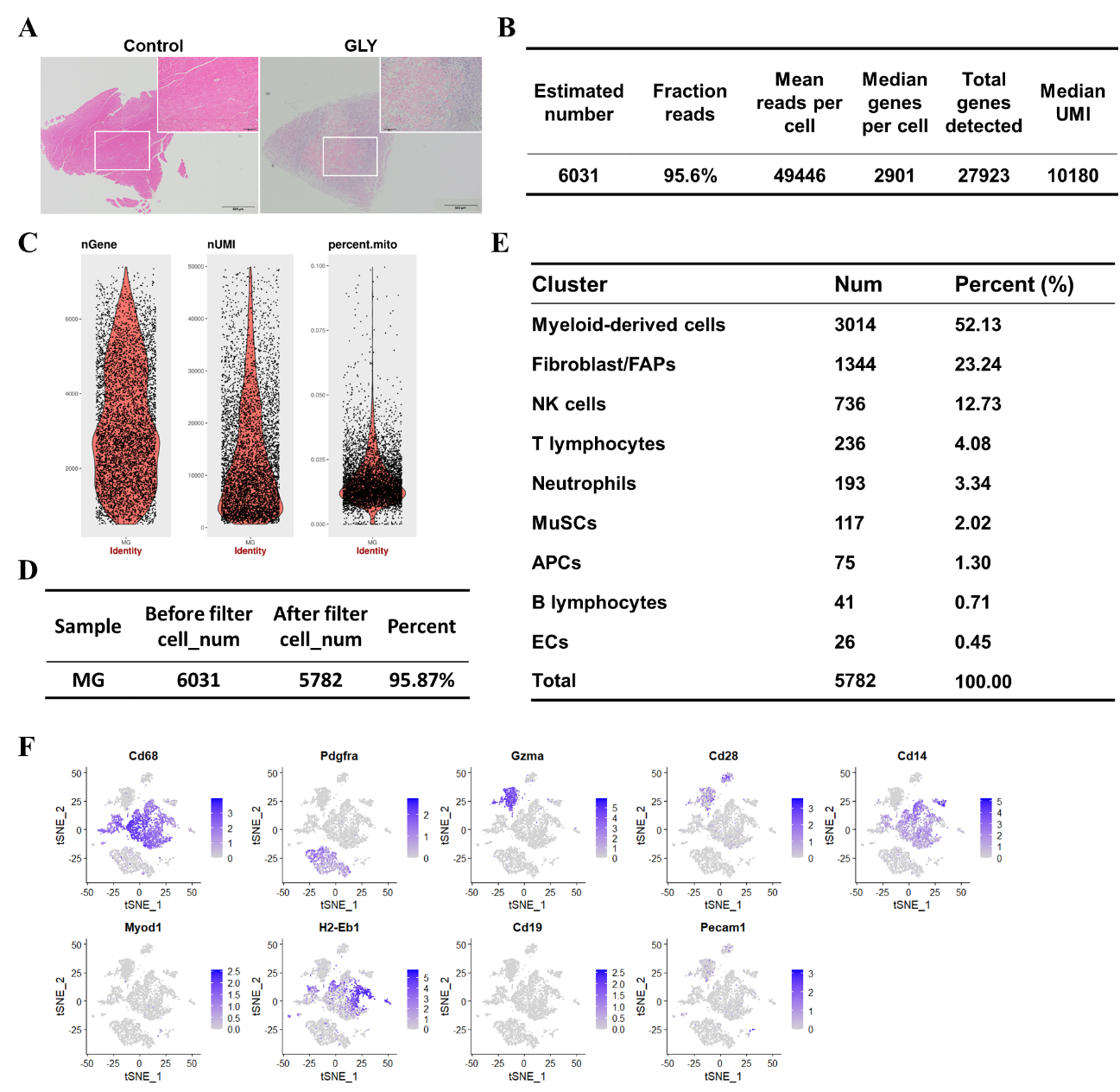
**

**Figure S2**


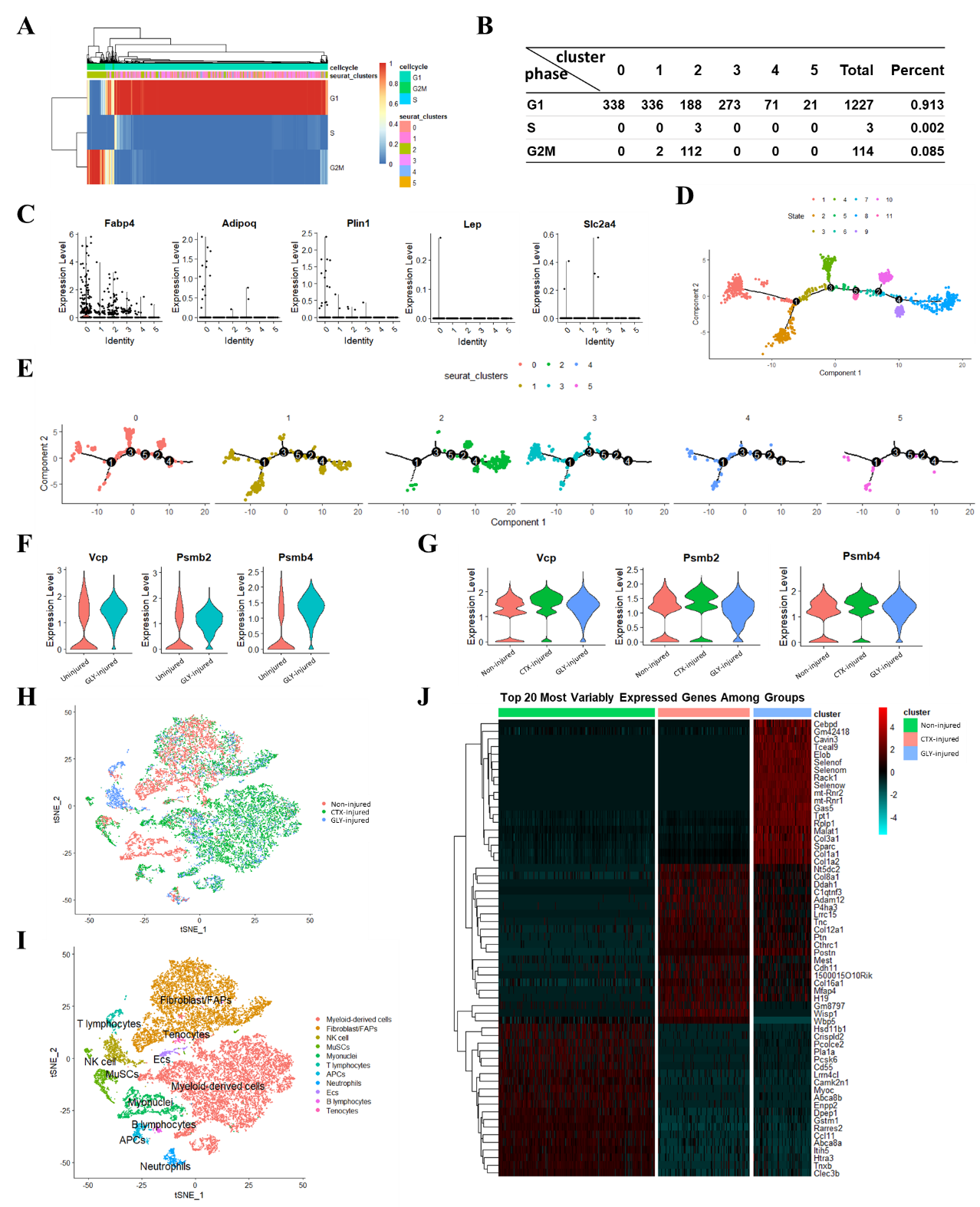


**Figure S3**

**
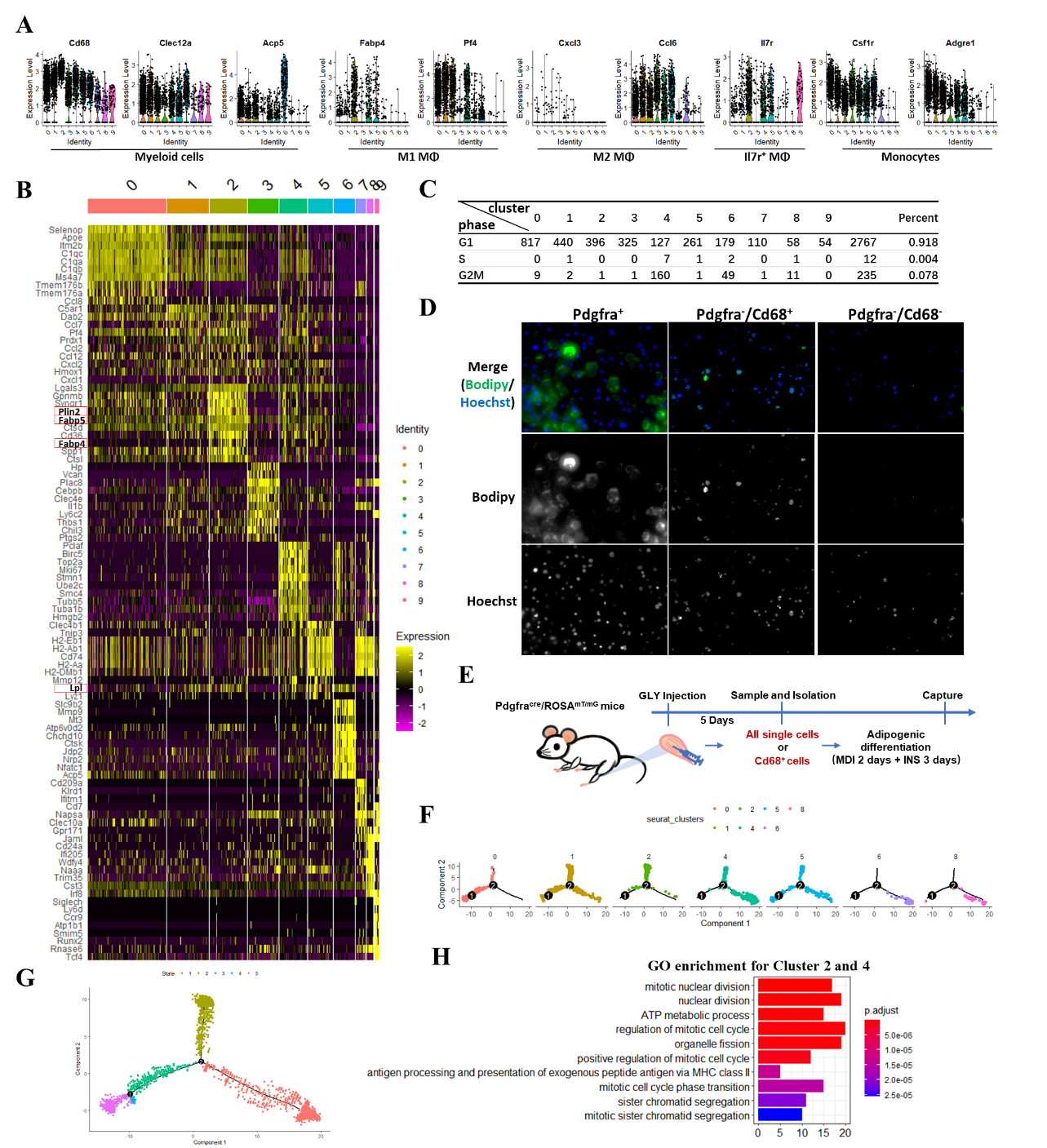
**

**Figure S4**

**
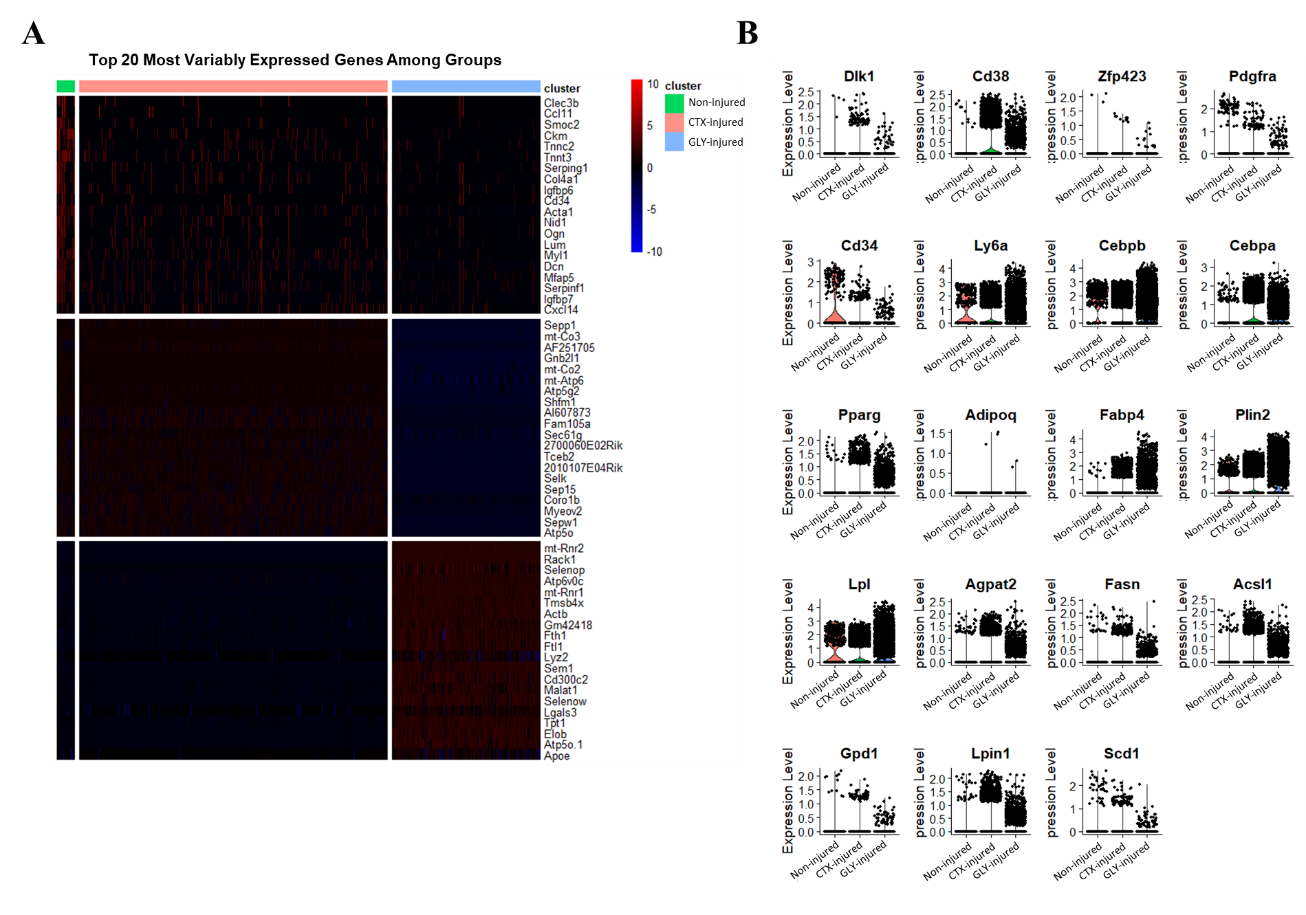
**

**Figure S5**


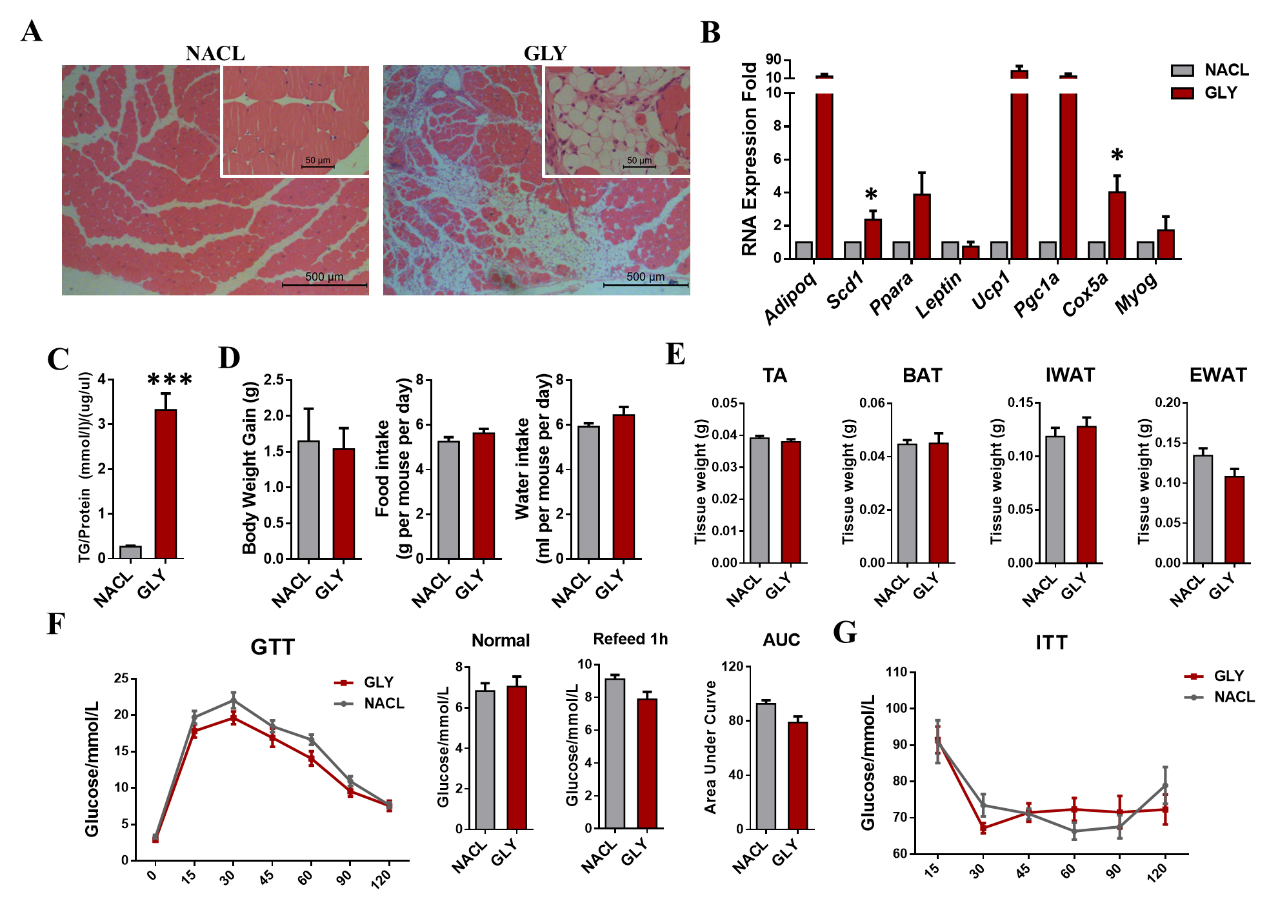


**Figure S6**


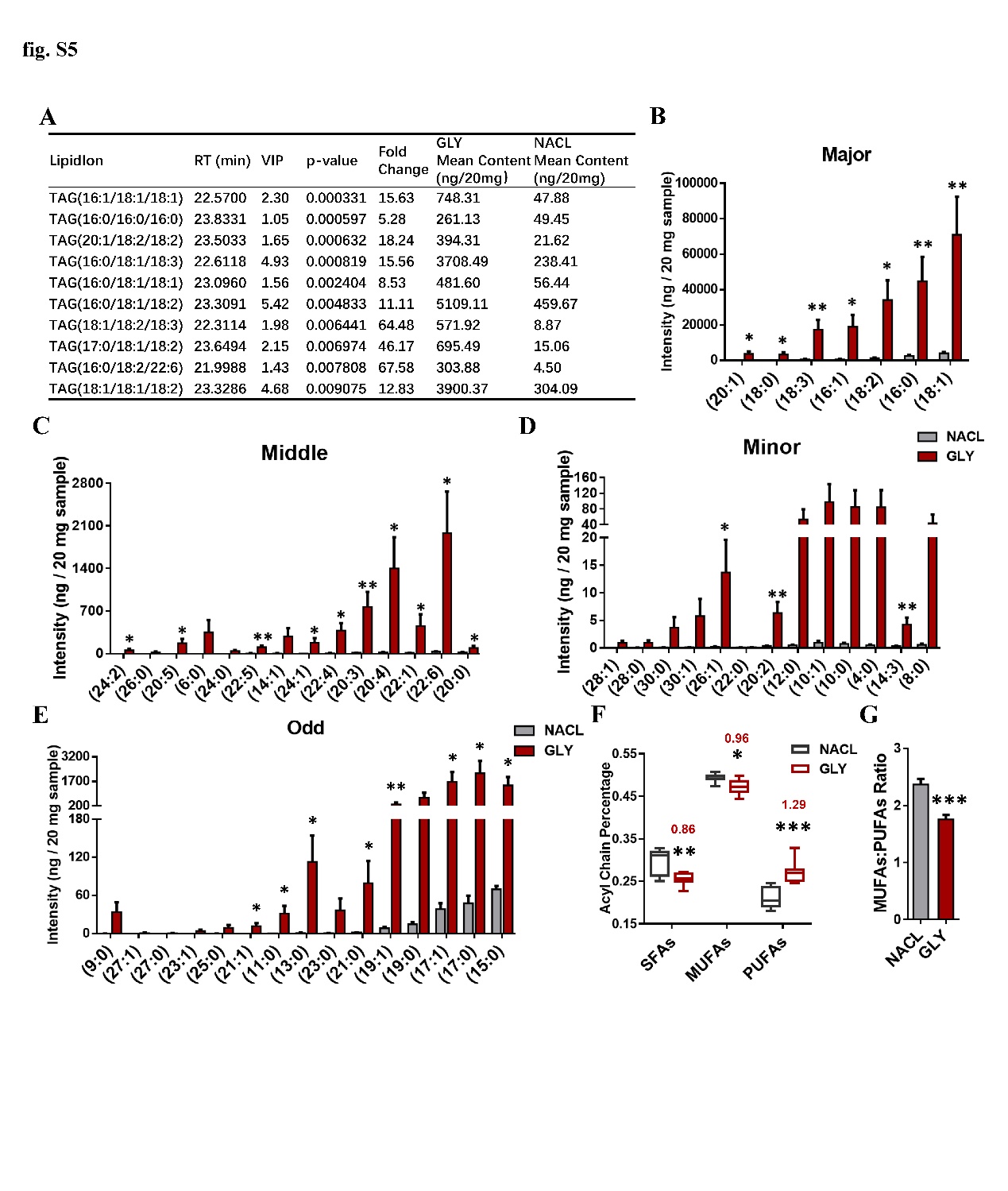


**Figure S7**


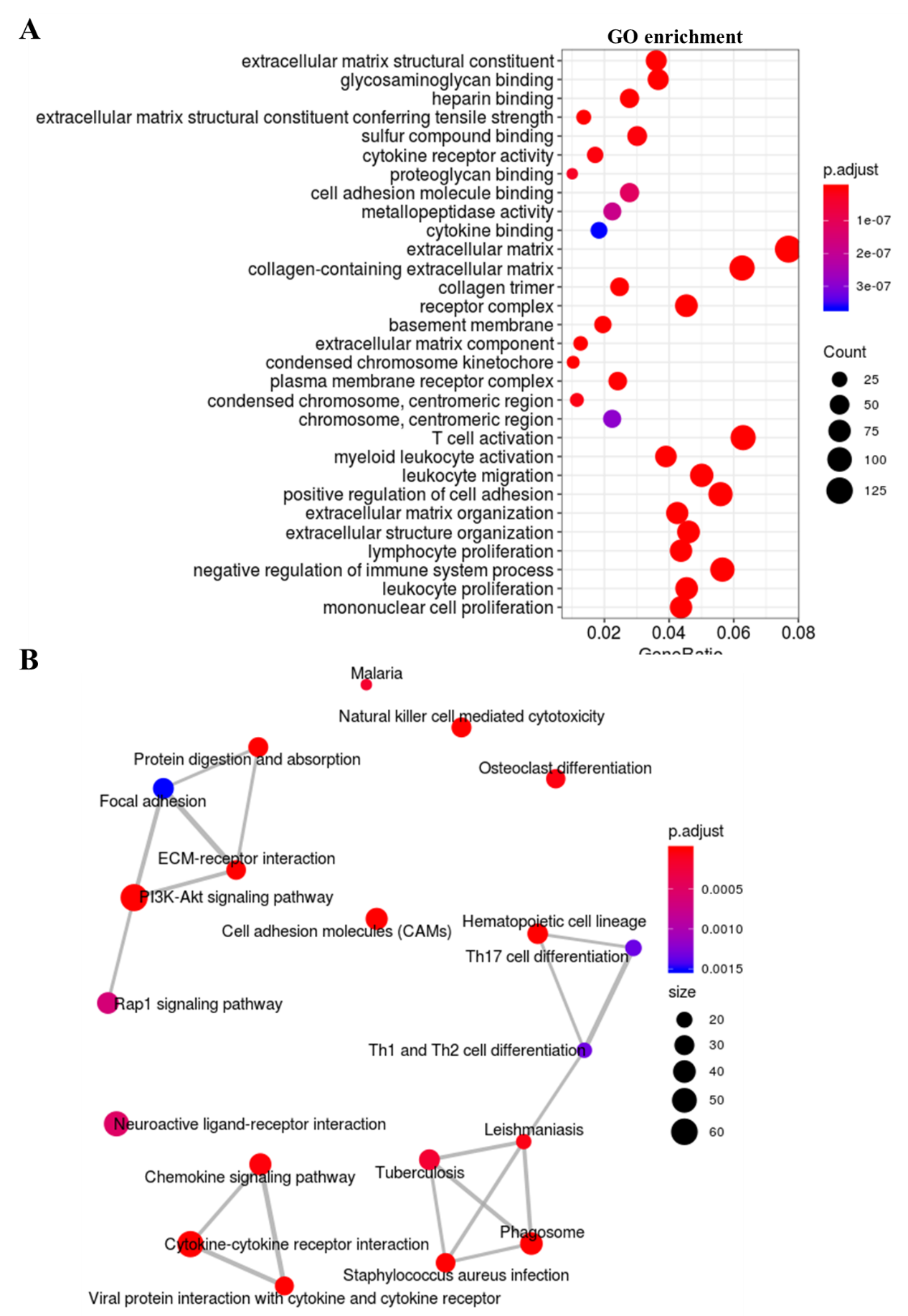


**Figure S8**


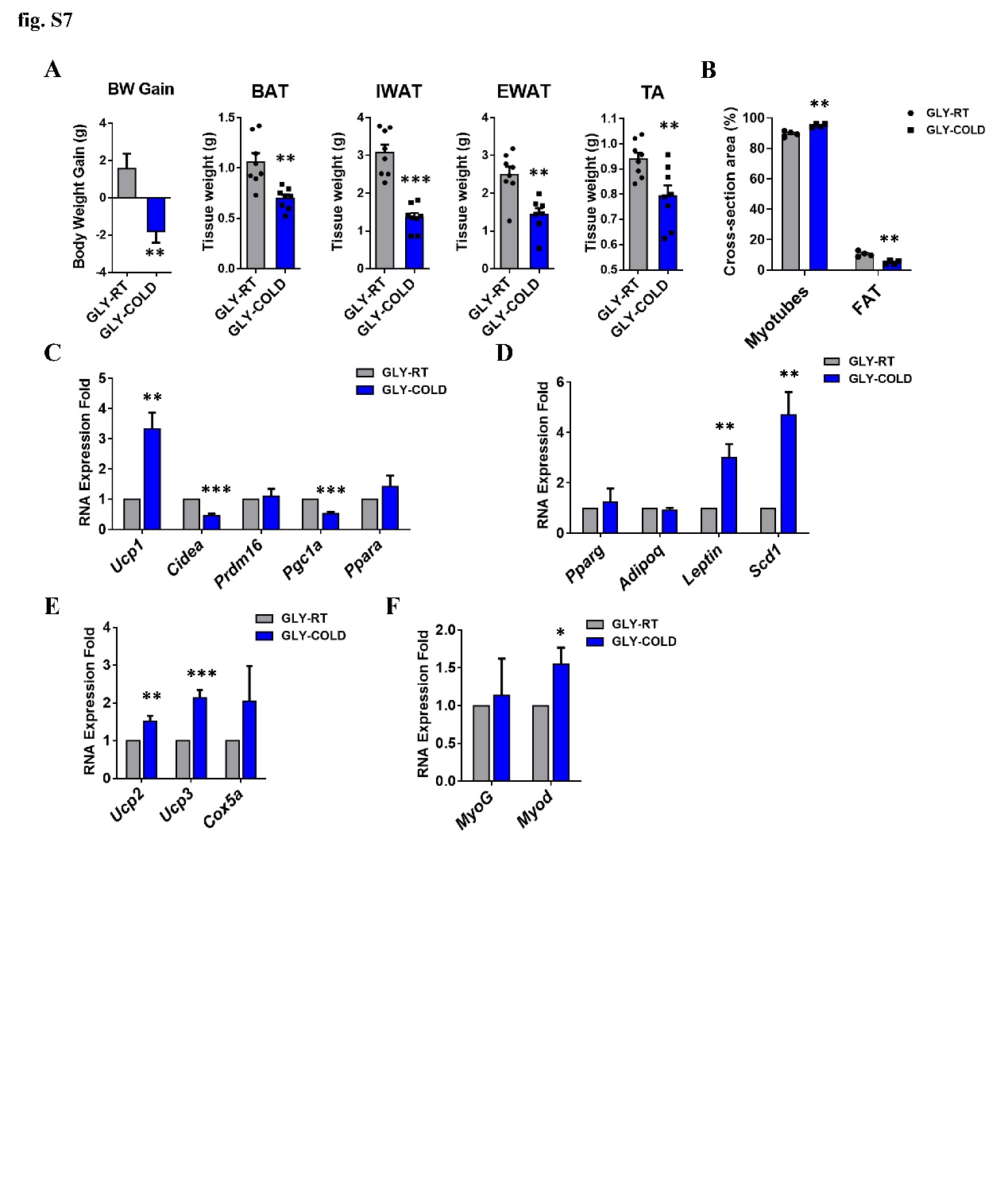


**Figure S9**


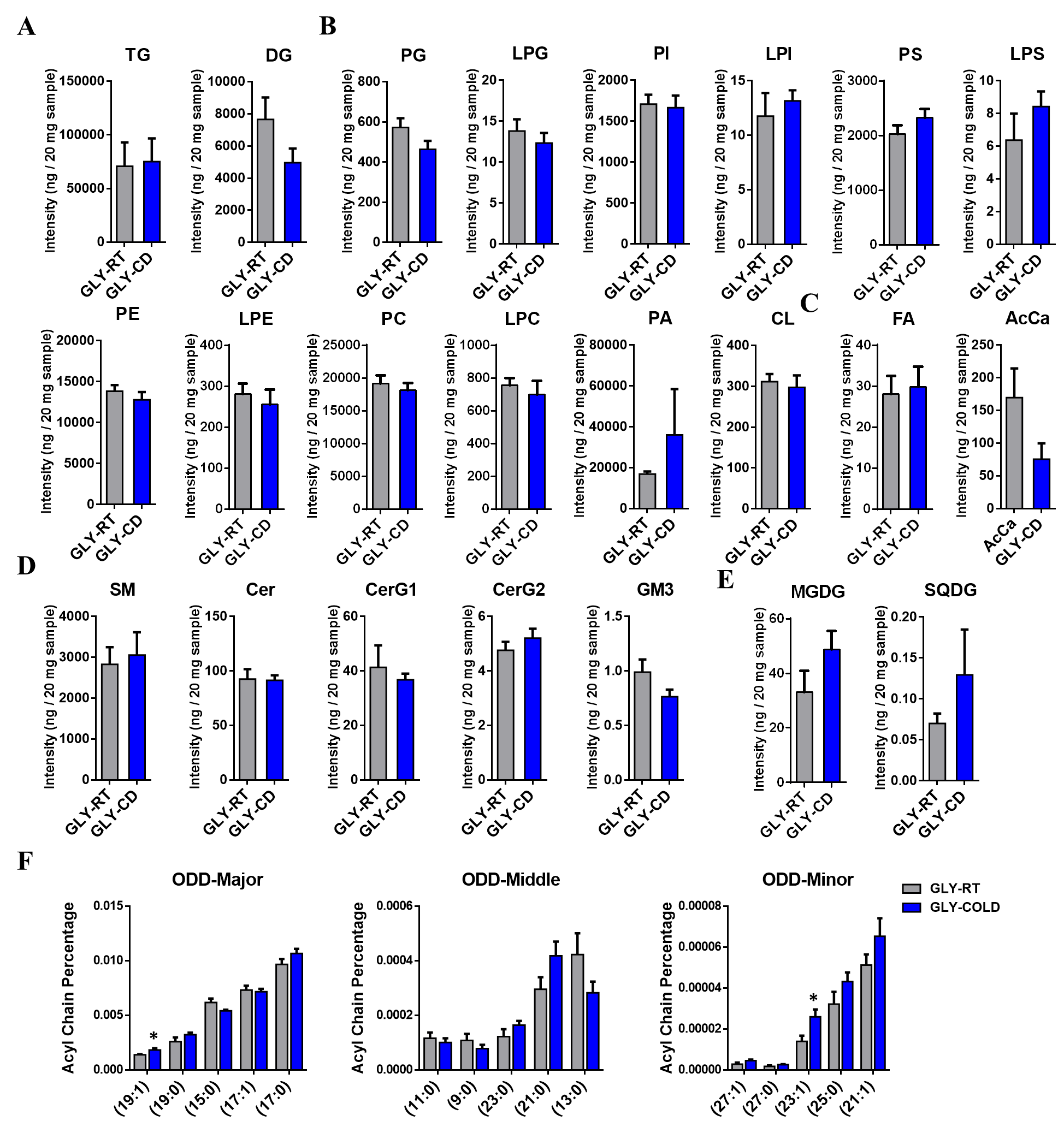


**Figure S10**


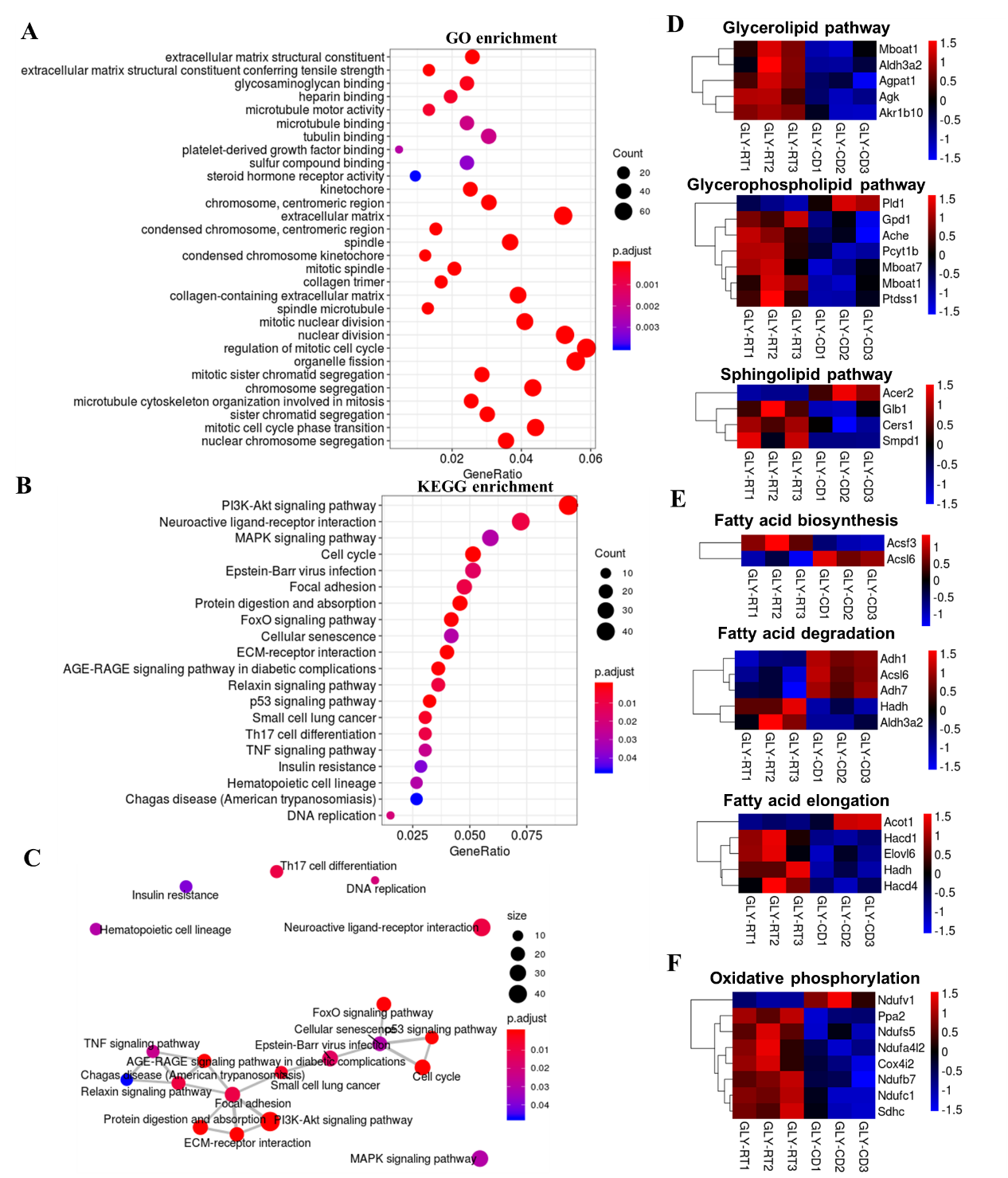

Supplement: Supplementary file 1 — Figure S1. scRNA‐seq identified distinct cell populations in GLY‐injured skeletal muscle. (A) H&E staining of control and GLY‐injected TA sections on DPI 5 (n = 3). Scale bars, 500 μm. (B) The results obtained from Cell Ranger analyses. (C) Quality control for scRNA‐seq datasets. (D) Cell number of scRNA‐seq datasets before or after filter. (E) Cell numbers of individual cell clusters. (F) Expression of representative genes in distinct cell clusters, including macrophage/monocytes (Cd68), myofibroblasts (Myl9), natural killer cells (Gzma), fibroblast/FAPs (Pdgfra), T lymphocytes (Cd28), neutrophils (Cd14), skeletal muscle stem cells (Myod1), CD4/CD8 T cells (Ccr7), B lymphocytes (Cd19) and endothelial cells (Pecam1). Figure S2. Clustering and pseudotemporal trajectories identify transcriptional dynamics of Fibroblast/FAPs. (A) Cell cycle analysis of myeloid‐derived cells. (B) Cell numbers and percent of fibroblast/FAPs in G1, S and G2/M phase. (C) Expression of mature adipocyte markers (Fabp4, Adipoq, Plin1, Lep, Slc2a4). (D) Pseudotime single cell trajectory reconstructed by Monocle2 for fibroblasts/FAPs. (E) Pseudotime single cell trajectorys for each subclusters of fibroblast/FAPs. (F) Expression of house‐keeping genes (Vcp, Psmb2, Psmb4) in new merged dataset of un‐injured and GLY‐injured skeletal muscle. (G) Expression of house‐keeping genes (Vcp, Psmb2, Psmb4) in new merged dataset of non‐injured, CTX‐injured and GLY‐injured skeletal muscle. (H) The t‐SNE plot of merged isolated single cells form non‐injured, CTX‐injured and GLY‐injured skeletal muscle. (I) Graph‐based clustering and cell types indentation of merged isolated single cells form non‐injured, CTX‐injured and GLY‐injured skeletal muscle. (J) Heatmap of top 20 significant genes between non‐injured, CTX‐injured and GLY‐injured fibroblast/FAPs. Figure S3. Clustering and pseudo temporal trajectories identified transcriptional dynamics of myeloid‐derived cells. (A) Expression of myeloid‐derived cells ma [file JCSM-12-109-s001.docx]
